# Analectic

**Published:** 1836

**Authors:** 


MISCELLANEOUS INTELLIGENCE.

### ANALECTIC, ANALYTICAL, AND ORIGINAL.

### I.—ANALECTIC.

*Ad Sylvas Nuncius.*

1. *Puerperal Convulsions.*—In the summer of 1833 I delivered a lady of her second child. The delivery was accomplished within an hour and a half from the first announcement of labor. Being hurried away to another case, I left her very comfortable, 30 or 40 minutes after delivery. About one hour and a half after, she was suddenly seized with a very violent convulsion, of that species described by Velpeau as *Apoplectic Eclampsia*. Before she could be administered to, another convulsion returned on the profound stupor and heavy stertorous breathing which followed the first. A consultation of physicians was present. The pulse being slow, but full and strong, liberal depletion was practiced. All the intermediate apoplectic symptoms continued notwithstanding. Soon after this second paroxysm, I arrived. Whilst receiving the above account, a third paroxysm supervened, which I witnessed, and which was very severe indeed—commencing during the stupor and stertorous breathing, full pulse, etc., by a drawing of the eyes and then the head to one side, until the body was universally convulsed, with frothing at the mouth livid skin, etc. This soon subsided, and was followed by the same apoplectic symptoms. On examination, notwithstanding the copious depletion from the arm, it was discovered that the uterine hæmorrhage was very copious indeed; the whole napkin, which had been but 10 or 15 minutes in application, being completely saturated, beside a large quantity of coagulated blood surrounding her in bed. On the external touch, the uterus presented no firmness; but the whole hypogastrium perfectly flaccid. The uterus was immediately grasped and agitated repeatedly through the abdominal parietes, until its contractions could be distinctly perceived to return with increasing strength, every four or five minutes. From the first application of the hand, no other paroxysm returned, and after about fifteen minutes from the commencement of this operation, the apoplectic symptoms disappeared, and she opened her eyes with intelligent expression, looked about as if surprised at seeing the family and physicians about her, and asked where was her child. It was brought, and she received it into her arms. She then asked for something to drink. A cup of weak tea was soon brought, which she drank sitting, spoke several times, and was again placed in bed.

I remained with her about an hour after, continuing the manual operation for exciting the uterus. Finding that she continued free from all alarming symptoms, I instructed her nurse to maintain the perpetual contraction of the uterus by the same process I had used, and left her, complaining of nothing but some painful sensations in the uterus at every contraction, amounting to slight after pains. The hæmorrhage had been reduced to the ordinary quantity for the period.

A few months after, I was present with another lady, who, after having had several convulsions of the same character, the first occurring in the latter part of the second stage of labor, was then suffering the intermediate apoplectic symptoms. On directing attention to the abdomen, the uterus was found in the same relaxed condition as in the former case. It was stimulated to action by the same means, and within from 20 to 25 minutes, the respiration, as well as the expression and color of the face, became natural; she opened her eyes with a natural expression, yawned, and turning her head to an easy position, slept well. It was found that she also had flooded very copiously. She had no more convulsions.

In view of the present state of the profession on this very interesting and important disease, I cannot withhold the facts of these cases from the public. They are given in the hope that in the present imperfect state of the pathology and treatment of these convulsions, the curative indication, in some cases at least, may be made so plain as to lead to more favorable practical results. What is the pathology of these cases? Was there a *passive* hæmorrhage from the uterus? And was there at the same time a strong general action of the heart, and a great determination of blood to the brain? And did the exciting means used with the uterus *equalize* the action of the whole system, by arousing the energies of this passive organ; and thus by a prompt and decided derivation of excitement, at once relieve the brain of its ruinous burthen? Or was the eclampsia a mere nervous phenomenon, arising out of the debility and consequent increased irritability of the motor apparatus; and the symptoms strictly apoplectic, only a secondary effect, arising from cerebral compression from blood forced into the brain by the violence of muscular contractions in this spasm? And did the excitement of the uterus so far employ the excitability of the system as to relieve other parts of that excess which predisposed to spasm? Would not ergot have produced the same beneficial effects as the manual operation employed, if it could have been administered? (yet it could not have been in either case.) May or may not the previous bleeding have prepared the system more perfectly for this counter-excitation? And would further bleeding have ever produced this equalization of action? Would not the patients have died without proper excitation of the uterus having been directly effected? May not the powerful antispasmodics which are, when they can be, almost always exhibited in these cases, operate, when they do operate beneficially, simply on this principle, (i. e.) exciting the uterus particularly, rather than the system generally? Would not tartar emetic have failed as well as continued depletion in these cases?

It is earnestly hoped, for the good of humanity, and the credit of the profession, that these facts and interrogatories may lead to more accuracy in pathology, and consequently, successful therapeutics.—*Southern Medical and Surgical Journal.*

2. *Carcinoma of the Tongue, successfully treated with the Ligature.*—In the American Intelligence contained in the American Journal for February, we have an account of a case reported by Dr. Donnellan, of Donaldsonville, Louisiana, of a large, ragged, ill-conditioned ulcer of the tongue, which, from its present character and the history of the case, he considered concerous: cured by the ligature, as recommended and practised by Sir Edward Home. The ulcer occupied the right anterior part of the tongue to a considerable extent, and was progressing very rapidly. The subject was a very corpulent women, 25 years of age, and in the 8th month of pregnancy, when he first saw her: at which time there was but a small ulcer on the part, apparently of no importance. On the 23d day after, on being called to the case, he found that the ulcer had made such fearful progress, that he deemed it proper to operate, and risk its effects on her pregnancy, in preference to allowing the ravages of the disease, even for the short remnant of the gestation period.

A crooked needle armed with a strong ligature was passed through the middle of the tongue behind the ulcer. The ligature was then cut at the needle, and one tightly drawn and tied on each side of the ulcer; thus cutting off the circulation from the diseased portion which constituted a considerable segment of the right side and tip of the organ. The pain on tightening the ligatures was very intense, but was soon relieved by 60 drops of Tr. Opii.

A few hours after the operation, a copious salivation supervened, which continued till the dropping off of the diseased part. Five days after the operation—29th April, deep sloughs were produced by the ligatures. On the 4th May, ten days after the operation, a single ligature was applied in the fissures caused by the old ones, so as to embrace the whole, which dropped off on the 6th—twelve days after the operation. On the 11th May, the vacuity was found fast filling up with granulations, and on the 23d, was perfectly cicatrized—the patient having given birth, some eight or ten days previous, to a fine healthy child. Her articulation was but slightly impaired, and at the last observation she continued in excellent health.—*Ib.*

3. *Sketch of the case of Dr. Crosby.*—By R. D. Mussey, M. D. Professor of Surgery, etc. in the Medical Institution, Hanover, N. H. Died at Hanover, N. H., Tuesday, April 12, 1836, Dr. Asa Crosby, æt. 70, of rupture of the gall-bladder.

This gentleman had been subject, for some years, to attacks of colic, with constipation. At 1 o'clock, on the morning of the Friday preceding his death, I was called to visit him, and found him laboring under pain of the abdomen, with costiveness. He told me that he had been feeble, with some pain in his bowels, for a few days, and that, in his opinion, a gall-stone was lodged in the bile duct;—he said that he had a similar attack last autumn, when, as he believed, a biliary concretion passed the bile tube after having been lodged there for some days. He had now taken, within a day or two, some cathartic pills, such as usually suited him well, but they had not operated. He was somewhat alarmed by a strong rigor, with but little sensation of cold, which had attacked him half an hour before I saw him, and which had not entirely subsided, although there was no sense of coldness remaining. There was tenderness of the epigastrium. I prescribed enemas, which gave him pretty clay-colored discharges, with considerable relief. On Thursday the family had observed a degree of yellowness of the skin and conjunctiva. This was more vivid on Friday. During this day, a diffused tenderness over the abdomen was manifest, and the bodily strength declined. On Saturday and Sabbath, these symptoms increased, notwithstanding the bowels were opened without much difficulty. A free bleeding brought temporary relief and exhibited buffy blood, but the strength gradually failed, until Tuesday morning at 6 o'clock, when he died. The yellowness of the skin slowly faded after Friday, and was very slight at the time of death.

*Post-mortem appearances.*—A portion of the abdominal peritoneum and that of a part of the small intestines were slightly adherent, with a thin layer of lymph interposed. Several coils of small intestine moderately reddened; half a pint or upwards of serum in the peritoneal cavity. A deep yellow color was extensively diffused over the right side of the abdomen; the gall-bladder, covered with fat, was empty and shrunk, and on opening it, a perforation of its coats, upon its hepatic surface, was observed, one third of an inch in diameter. A great deal of fat surrounded the kidneys. On the right side of the abdomen this was colored with bile, and from the root of the liver to the lower part of the right iliac region a very large quantity of bile was deposited, entirely *behind* the peritoneum—this membrane not having been ruptured.

In the common biliary duct, a gall-stone, of the size of a large pea, was fixed at the distance of about an inch from the intestine. This duct was very small between the gall-stone and its outlet, but from the gall-stone to the gall-bladder it was dilated sufficiently to admit the little finger. No other gall-stone was found in any part of the biliary apparatus.

There was nothing worthy of remark in the chest, except perhaps, a slight thickening and shortening of the mitral valve. In the brain nothing was observed, except the size of the medulla oblongata, which was smaller than I recollect ever to have seen it in the adult brain.

There can remain no doubt as to the cause of death in this case, viz. inflammation from affected bile; the effusion occasioned by rupture of the gall-bladder from an obstruction of the common duct. Did the rupture take place about the time of the accession of the rigor on Friday morning?—*Bos. Med. and Surg. Jour.*

4. *Cold Baths in the treatment of Chorea.*—Chorea is one of those diseases of whose seat and nature we are entirely ignorant, and, of course, whose treatment is entirely empirical. An enumeration of all the therapeutic means which have been from time to time extolled for its relief, would nearly comprise the whole materia medica. Of all the curative means, the bath, either simple or medicated, is that at present most generally used in France, and appears to merit the most confidence. It was a favorite remedy with Dupuytren, who said he had never seen a case of St. Vitus's dance resist the bath by immersion. His method of administering it was, to cause the patient to be held by two persons, one having hold of the arms, and one of the feet, and then the whole body to be immersed in the water, and immediately withdrawn, which operation was repeated seven or eight times in the space of ten to fifteen minutes. After this operation, the patient was carefully wiped dry, and either taken to bed or required to take half an hour's violent exercise. With females, Dupuytren was contented to employ simple cold affusions to the head. This method was not without its inconveniences. Cerebral congestions are apt to be produced if the head is not immersed at the same time with the rest of the body; and thoracic phlegmasiæ have been produced by it. At the *Hospital des Enfans*, a simple cold bath is used; and this is not used in severe weather. The patient remains in it about an hour. If cold affusions are judged necessary, the patient is placed in a tepid bath, the chest enveloped in a pelerine of gum taffeta, and cold affusions are then made to the head. The regimen is always tonic, the patient taking at each meal a certain quantity of wine. Three striking cases are related by T. Constant, as having occurred at the Children's Hospital, which strongly attest the efficacy of the cold bath in *Chorea St. Viti.—Bulletin Generale de Therapeutique, Medicale et Chirurgicale.—Ib.*

5. *Examination of the Head of Fieschi, the French Assassin.*—The cranium presented exteriorly the marks of two wounds, one of which was situated over the posterior-superior angle of the left parietal bone, and occupied a considerable surface; the other, much smaller, was seated a little above the extremity of the left eyebrow. Near the angle of the mouth, on the left side, was an oblique cicatrix, nearly half an inch in length, so completely formed, that it might have passed for the mark of an old wound. The external wound of the integuments, above the ear, was not yet closed.

The soft parts were now carefully removed by a crucial incision, and the bone exposed. At the point corresponding with the wound in the left parietal bone, was observed an oval projection of the osseous wall, as large as a crown-piece, this prominent portion of the bone was perfectly circumscribed, and bore some resemblance to a watch-glass fixed in its circle. Its surface was throughout uniformly convex, except at a small point inferiorly. On cutting through the skull, and examining its internal surface, a concave space of the same form and dimensions corresponded with the external projection, and it now became evident that the latter was nothing else than a portion of the skull, which had suddenly and in totality been removed, as it were, by the action of a punch, and being at once replaced by the surgeon, had subsequently united to the rest of the cranium. A very thin false membrane lined the internal surface of the fractured bone, and separated it from the dura mater; however, the membranes of the brain near the wound were perfectly healthy, and presented trace neither of external lesion, nor of inflammation. It was evident the brain had not been injured, a circumstance which explains how the assassin was able to descend from his chamber-window by a cord into a neighboring court; yet the shock must have been dreadful. The wound on the forehead seemed only to have entered the external wall of the frontal sinuses. Fieschi, during his trial, spoke of seventeen or twenty fragments of bone which had been removed from his head; this was an exaggeration, if not false, for nowhere could any loss of substance be perceived.

The nature of the wound, and particularly its mode of union, offer several particularities of the highest surgical interest. No doubt M. Lelut will shortly publish a detailed account of the autopsy.—*Lancet.—Boston Medical and Surgical Journal.*

6. *Double vision.*—Two cases of double vision are related in a foreign medical journal, in both of which, at a certain distance from the eye, two distant objects were seen, one a few inches above the other. One was in a musician, a young man of 22, of abstemious habits, and who had applied himself assiduously to playing and writing music, being often thus engaged a greater part of the night. It was thought, in this case that the habit of looking at two bars of music at once was the cause of the affection. The other case was first noticed, by a gentleman while traveling in the south of France. There was an uneasiness in the head, a confinement of the bowels, and, in the left eye, a yellowish spot as large as a pea was noticed, although in this case, as well as in the other, both eyes were equally affected. In two months, the patient having been purged and cupped, and having also returned to his previous habits of active exercise, the affection wore off.—*Boston Med. and Surg. Jour.*

7. *On the Modifications of Structure in the Thorax and Lungs, produced by Age.*—By MM. Hourmann et Dechambre.—1. *Thorax.* There are two different conditions. (1.) A certain number of old women retain a fresh appearance, color in their cheeks, suppleness of the skin, few, and not deeply-marked wrinkles. Their breasts are voluminous, generally pendant, but sometimes firm. The whole thorax is covered with a layer of fat, the muscles are well nourished and vividly red, the cartilages of the ribs retain some of their whiteness and elasticity; in some cases not one is ossified. There is but little change in the texture of the sternum and ribs. In some cases there is some lateral flattening of the upper part of the chest, augmenting the antero-posterior diameter at the expense of the transverse, so that anteriorly it appears very narrow between the armpits. The base however is large, so that it looks like a truncated cone. Sometimes there is another contraction, which is less marked, about the level of the eighth rib. Both contractions, however, are more evident in the second form.

(2.) This is the most frequent; the other evincing a vigorous condition, which is an exception. The lateral and superior flattening is carried to an extreme degree, as has been noticed by Soemmering. The posterior curve of the ribs is consequently increased, forming a marked round or angular prominence on each side of the dorsal spine. When unequal and on both sides it is often attributed to lateral curvature; and when unequal on one side, forming an uneven surface, it impedes immediate auscultation. From the mobility of the sternum it is carried forwards, so that the anterior part of the chest looks as if formed by two inclined planes, meeting in front, with the angle of union truncated. Another deformity is owing to the use of stays, which by contracting the base of the thorax give it the form of a small barrel. In many cases, however, the contraction is three or four fingers' breadth above the lower margin of the ribs: so that the margin itself instead of being thurst into the abdominal cavity, is turned outwards, and the edges of the last cartilages form a marked prominence beneath the soft parts. This alteration of form is important, from the changes it produces in the viscera beneath. The liver instead of being always pressed up towards the chest, is, on the contrary, thrust downwards into the abdominal cavity. The portion beneath the stricture is found strongly applied to the upper surface of the liver, which often bears the mark of the pressure that it has suffered on a level with the contraction. The right lung is not pressed up towards the summit of the chest, but is elongated by following the liver in its descent, so that there is not the usual difference in the length and volume of the two lungs. The sternum is forced forward, but as the clavicle and first rib prevent its motions superiorly, the xiphoid cartilage is pressed backwards, and is sometimes overlapped by the cartilages of the last true ribs, which are brought so near together as sometimes to cross each other. (See Cruveihier's Anatomy, vol. i.) This force, acting in contrary directions at each end of the sternum, produces a kind of separation of the two upper pieces of which it is composed, and an arched eminence at the point where the diastasis takes place. Soemmering has remarked this, as well as the change in the relation of the anterior planes of the thorax and pelvis. They no longer correspond, but the former exceeds the latter; the contrary being the case with old men.

The dimensions of the chest are also changed in the longitudinal direction, from a diminution in the heighth of the intervertebral substance. Fischer mentions the case of a man, æt. 100, in which nine of the vertebræ were reduced to a complete colume of bone; and the same change was observed by Boerhaave in the whole length of the spine; our authors have seen very commonly three or four vertebræ thus united. Haller attributed this to the absorption of the intervertebral substance, and Morgagni to its ossification; both admit, however, that the spine is shortened. The absorption is more common, than ossification. As the muscles from age become unable to maintain the trunk erect, it bends forwards; and the flat surfaces of the bodies of the vertebræ press strongly against the anterior part of the disks which separate them. In this way (as Seiler observed) union takes place, rendering the inflexion permanent. The last cervical and first dorsal vertebræ are most bent. In some the cervical region makes almost a right angle with the dorsal, and the chin rests against the sternum. A curve in the opposite direction occurs in the lumbar spine, the convexity of which pushes the base of the chest forcibly forwards. From this shortening and curve of the spine, the interval is diminished between the inferior margin of the thorax and spine of the ilium, and also the ribs approach each other more nearly, especially in front. If it is also recollected that from the lateral flattening of the thorax, the ribs are so twisted that their external face is turned directly, instead of obliquely outwards, so that their edges are placed perpendicularly one over the other, the contracted state of the intercostal spaces will readily be conceived. Seiler has also noticed as a consequence of this bend, the elongation of the extensor muscles, and the contraction of the flexors, which contraction becomes permanent; as is seen particularly in the sterno-cleido-mastodei, which appear like cords when the head is a little raised.

The textures covering the thorax are atrophied; the mammœ and fat have disappeared, and the muscles are very thin. The skin is thin, rough, dry, and of a dirty brown color. The form of the muscles is seen distinctly. The diaphragm, as may be imagined from so many changes in the form of the thorax, is thrown into folds, which make impressions on the liver. The parts which compose the sterum are united; its cells are large, and often filled with a reddish pulp. The ribs are also less dense, and have lost their elasticity. The cartilages of the first and second ribs are ossified, but it is rare to find bony incrustations on the others; when it takes place it begins in the centre. Union of the chondro-sternal articulations is rarer than is imagined; the costo-vertebral joints generally preserve their mobility.

2. *Lungs.* The lungs of the old are variable in their aspect. The varieties may be comprehended in three typical forms, by which one lung may differ from another, or parts of the same lung from other parts. Magendie has attended to this subject.

1st. Type; or the lungs of muscular, stout, vigorous old women, with a capacious thorax. The external aspect differs very little from the lungs of an adult; except in the direction of the great interlobular fissure, in those cases where there is lateral flatness of the thorax. This fissure in the adult has the upper lobe lying immediately above it, and passes obliquely to the root of the lungs, so that on the right side the central lobe occupies exactly the middle part; and on the left, has the lower-lobe immediately beneath it. But in old age the fissure becomes vertical, so that one lobe of the left lung is directly in front and the other behind, and the middle lobe of the right lung projects downwards, and the lower lobe becomes elevated behind it, so as to form the posterior fourth, or even more, of the summit of the organ. Thus pneumonia of the summit may be seated in the inferior lobe.

2d. Type. The lungs are of regular form, but small, light, and hardly capable of being distended sufficiently to fill the thorax, even by the strongest inflation. They are bathed in limpid serum. Heart small, thorax contracted, soft parts emaciated.

3d. Type. Lungs forming a mass, of which the surface is irregular, pressed close to the spine, and surrounded by much serosity. They are livid and flabby, have lost their conical form, the summit being often larger than the base. The lobes are sometimes merely united by a flat thin pedicle, which leaves them as it were floating: the fissures have disappeared. Inflating them does not much increase their size. They are very light, and give to the touch the sensation of a skein of flax. Heart small, often anæmic; and the thorax often excessively emaciated.

*Intimate Structure.* To examine this the lungs were simply dried, and not previously inflated, for obvious reasons.

1st. Type. A thin dried section appeared full of rounded holes, approximating like those in lace. Their diameter was about a quarter of a line, and each was perfectly isolated and distinct. The pulmonary tissue was divided and subdivided very minutely by linear tracts, which were seen by a glass to be evidently vascular.

2d. Type. A similar section was made: the cells were not round but elongated into ellipses so as to look like a series of chinks, sometimes a line in length, and terminated by two commissures more or less angular. The vascular tracts were equally elongated, and less numerous. The circumferences of the cells were distinctly seen and isolated.

3d. Type. The cells were of no distinct form: a section could only be compared to torn net-work, whose debris intercepted the irregular spaces. But very few small vessels could be seen by a glass, and there was no division into lobules.

Such was the appearance in those who had not had symptoms of disturbed respiration, and so far they may be considered normal. This natural emphysema is more clearly shown when such sections are compared with others from the lungs of adults. In an adult the cells are at least only half the size. In Type 1, the cells were one fourth of a line; in an adult, they were one eighth, or at most one sixth of a line; and in children of four to six years old, about the twelfth of a line, finally, in a newly born infant, they were no larger than holes made by the finest needle. These facts verify the law announced by Magendie, from a comparatively superficial examination, that the density of the lungs diminishes, together with the quantity of blood they admit, with the progress of age. The thorax itself is gradually accommodated to this change; it becomes atrophied as the lungs atrophy; it contracts as they contract; and the diminution of their vascularity, which is always in relation to the diminution in texture, shows the direct proportion between the weakened chemical power and the dimished mechanical forces. The effusion of serum may be owing to the thorax being unable to contract beyond a certain point, so that as the lungs still diminish this may fill the vacancy.—*Archives generales de Medicine*, Aout, 1835.—*British and Foreign Medical Review.*

8. *On the effects of Nux Vomica and Aconite on Animals.*—By Dr. Lombard, of Geneva.—Dr. Lombard, while employing these remedies in his clinical practice, thought of trying their effects on animals, and made some experiments accordingly, chiefly in reference to their action on the heart. The experiments were performed on frogs, whose hearts beat with great regularity, and for a considerable time after the animal had been mutilated. The medicine was intoduced into the stomach, or applied locally to the heart, which was laid bare after the animal had been stupified by blows on the head.

1. *Nux Vomica.* When introduced into the frog's stomach, it produced tetanic convulsions, which in a few hours caused death. The contractions of the heart were sometimes strong and complete, sometimes irregular, tumultuous, and intermitting; always diminished in frequency. Applied locally to the heart, it slightly stimulates, rendering the pulsations more energetic and frequent.

2. *Aconitum.* Its internal employment renders the pulsation less frequent, without irregularity. It has, therefore, a decidedly sedative effect on the heart.

From these experiments, M. Lombard ventures to predicate the effects of the same remedies on the human body, and thence deduces the following practical inferences:

1. That nux vomica cannot be used with advantage in any diseases of the heart; for, although it diminishes the frequency of the pulsations, it renders them irregular.

2. That the aconite, being decidedly sedative, is a proper remedy in active diseases of the heart.

This effect of aconite has been observed on the human subject. In cases of poisoning, the contractions of the heart have been found diminished, and almost suspended. (Orfila, *Toxicologie*, t. ii. p. 221.) It is also considered by the homœpathists to be be an energetic antiphlogistic. On these grounds, M. Lombard has tried it in acute enteritis, pneumonia, etc., with success, and also in hypertrophy of the heart. He has often found some drops of the tincture diminish the frequency of the pulse, but more frequently diminish its strength and hardness.—*Gazette, Med. de Paris*, Oct. 10, 1835.—*Ib.*

9. *Pathology of Rheumatism.*—By M. R. Parise.—[This paper is written to prove that rheumatism is an irritation either of the large nervous trunks, or their ramifications, or their intercellular expansions, or the fibrillæ running among the muscles; that is, one of the neuroses. The author does not include articular rheumatism, which he regards as a simple inflammation of the membrane lining the joints. He has suffered from rheumatism in his own person, and as this does not deprive the sufferer of his mental powers, or interfere with their activity, we may admit this claim to attention. His arguments are—]

1. That pressure does not increase the pain, but often diminishes it. It is striking how the slightest notions of a limb may increase the pain, whilst compression relieves it.

2. It does not terminate in appreciable organic lesions, even though it should last for months or years. The muscular and nervous tissues are unchanged: there is never supuration, and it is doubtful whether serous or gelatious effusions beneath aponeroses, or the sheaths of tendons, is the result of rheumatism which has preceded them.

3. Its mobility is a distinguishing character.

4. Its change of situation does not change its nature, although it goes by different names. Thus it is the same disease which in the head is called *gravedo*, in the neck *torticollis*, (wry-neck,) in the side *pleurodynia*, in the loins *lumbago*, and along the sciatic nerve *sciatica*.

5. Rheumatic pains are more severe in the night, which is a character of neuroses. Heat cannot explain the fact, for the same heat during the day has no such effect. The cause is hidden.

6. Rheumatic parts are affected constantly by changes in the air and by electricity; the sufferers are living barometers; and this can be explained by the anormal sensibility of the nerves.

7. The distinguishing characters of the neuroses are, the irregularity of their progress, a tendency to periodicity, a disposition to cease and to return rapidly. These features distinguish rheumatism.

We shall offer no apology for not translating the following pertinent jeu d'esprit from one of Madame Sevigne's letters to her daughter, as it is too "spirituel" to bear translation. "Devinez ce que c'est que la chose du monde qui s'en va le plus vite et qui s'en va le plus lentement; qui vous fait approcher le plus de la convaelscence, et qui vous en retire le plus loin; qui vous fait toucher l'etat du monde le plus agreeable, et qui vous empeche le plus d'en jouir; qui vous donne les plus belles esperances, et qui en eloigne le plus l'effet; ne sauriez-vous le deviner? Eb bien! c'est un rheumatisme."

8. The facility of return after its apparently complete cure is so constant, that adults, and still more old people, never are sure, after having been several times affected, that they are permanently cured. Hence the rheumatic diathesis. Young people are more often completely cured; which is probably owing to their perspiring more freely and generating more heat, from a more active capillary circulation. This becomes more languid in advancing years; and explains the greater tendency to rheumatism in the old; in different seasons and climates; and the action of flannel, rubefacients, etc., in its cure.

9. Finally, it is often cured by antiperiodics, as bark; but M. Parise proposes to discuss its treatment on other occasions.

The obscurity which hangs over rheumatism renders ever attempt at its elucidation recommendable. The similarity between rheumatism and neuralgia is clearly stated by M. Parise, but his opinion that articular rheumatism is a pure and simple inflammation of the membrane lining joints, going through the usual stages of inflammation, and requiring a similar treatment, is too hasty an assertion. If this were the case, there would be no difficulty in the matter; but the circumstances of its not yielding, like other inflammations, to treatment, its not producing the same rapid disorganization, its rapidly shifting its situation, the facility of its return, and the difficulty of a complete cure, mark its specific nature, and have led pathologists to class it with the affection which M. Parise has described.—*Bulletin general de Therapeutique*, Julliet, 1835.—*Ib.*

10. *On the use of Purified Alumina in Infantile Cholera, and of Sulphate of Copper in softening the Stomach.*—By Dr. G. E. F. Durr.—Dr. Durr has been in the habit of using the *Argilla depurata*, in the dangerous forms of diarrhœa and cholera, which are liable to attack children especially during summer, with considerable success, since the year 1826. Its action appears to be very different from that of the carbonate of potass or magnesia. It may be given with perfect safety during the first stage of the disease, when on account of the inflammatory symptoms the muriate and carbonate of iron and opium are contra-indicated, and yet where the rapid course of the disease requires decided and active treatment. It has acted successfully in cases where these astringents and also chlorine water have failed; and Dr. D., prefers it to the acetate of lead, which at this early age is rather a questionable remedy. To use the argill successfully, we must give it in pretty large doses, viz. from half a drachm to one drachm, in any proper vehicle, during the twenty-four hours.

The chief symptoms of this dangerous affection, which runs its course in from two to ten days, are profuse vomiting, without any effort, of a sour smelling fluid varying in consistence; in many cases diarrhœa had lasted a whole week, when the first alarm was excited by the sudden appearance of vomiting. Collapse and rapid emaciation of the body followed, with depression of the anterior fontanelle; hollowness of the eyes, paleness, alteration and shrinking of the features, cold extremities, hot occiput, and more or less fever; *agrypnocoma*, or a lethargic state without actual sleep, restlessness, crying, whining, throwing itself from one arm of the nurse to the other, drawing up the feet to the abdomen, want of appetite, great thirst, stiffness in the nape of the neck, and the stomach so distended that it projected in the left hypochondrium like a distended bladder.

Dr. Durr's practice in this disease was to quiet the irritation in the stomach and bowels by emollient oleaginous remedies in combination with the argill: to excite the activity of the skin, by extr. cicutæ internally, and the application of an epipastic powder externally. The immediate effects of this were diminished frequency of the evacuations, the natural yellow color returning, quiet, excoriations in the folds of the skin about the neck and groins. In very young children the cerebral affection was often allayed merely by the chlorine water (aqua oxymur.;) in older children, or where the symptoms were more violent, by leeches to the scrobiculus cordis, or behind the ears, according to circumstances; the dryness of the skin, the lethargy, and coldness of the extremities, were treated with baths of chamomile and salt, and with cold lotions to the head; warm stimulating aromatic fomentations were used from time to time, and enemata of elder and linseed, to which the yolk of an egg rubbed down with linseed oil was added. Dr. D. assures us that in other acute diseases also, where the rough dry state of the skin and defied the usual remedies, gentle perspiration had followed the use of these enemata. The epispastic powder which he mentions was first described by Autenrieth; it consists of fresh prepared mezereon bark powdered. When the skin is not very delicate, it not unfrequently fails to produce any effects. Dr. D. has used it combined with calomel, and in very severe cases combined with corrosive sublimate, with great certainty and effect. The spot to which it is applied usually becomes red in the course of from six to twelve hours, and in about as much more time moist and excoriated. If the powder will not stick, he moistens the spot with a little saliva or lard.

The result of his practice is decidedly favorable; of 67 children from the time of birth to the age of fifteen months which he has treated for this disease during 1833 and 1834, he lost only seven. Dr. Durr has given several interesting cases, both successful and unsuccessful, together with the examinations of the latter after death. Great congestion of the cerebral vessels, and considerable softening of the stomach, so that portions of it were quite pulpy, were the chief features, and in one case there was perforation. We regret that our limits will not permit us to quote more than one of these.

*Acute Softening of the Stomach.*—A weakly but apparently healthy female infant, eleven days old, brought up on saccharum lactis. The parents lost a child at the same age in August 1833, with symptoms of a similar description, in three days. The child sickened rapidly (Aug. 1834,) with diarrhœa of clay-colored evacuations; constant screaming, restlessness, red tongue, hot skin, distended stomach. ℞ Ol. Amygdal., Mucilag. Acaciæ, aa ʒ ij. Syrupi Altheæ ℥ ss.

Argillæ depuratæ, ʒ ss. Aq. Lauro Cereasi, ℈ ss. M. sumat cochl. min. j. quaque hora. The child recovered.

In the course of a week the same symptoms returned, with aphthæ and erythematous eruption over the whole body; the same emulsion was ordered with the addition of extr. cicutæ, gr. j. The child again recovered entirely. When about three weeks old (Aug. 24,) the diarrhœa returned with vomiting, drawing up the feet to the abdomen, screaming, restlessness, paleness of the face, and the eruption becoming fainter; the same mixture was ordered with the addition of Extr. Cicutæ, Rad. Ipecac. aa gr. j. Enemata, aromatic fomentations. Unguent. Cantharid. behind the ears, and the strong epispastic powder between the folds of the skin in the neck, exillæ, etc.

*2d day.* Little alteration during the night, except that the diarrhœa was not so frequent, eruption still scarcely visible. Stiffness in the spinal column; extension of the limbs; five green spinach-like evacuations; hands and occiput hot; eyes somewhat sunk; anterior fontanelle slightly depressed; slight convulsions; a half moribund look, alternating with screaming, during which the eyes become a little more animated.

℞ Mucilag. Acaciæ, Syrup. Altheæ, aa ℥ ss. Argillæ depurate, Liq. Ammon. Acet. aa ʒ ss. Pulv. Ipecac. gr. j. Ext. Cicutæ, gr. ij. M. sumat cochl. minimum quaque hora, contin. omnia.

*3d day.* Better during the night; bowels opened four times, yellow, not as watery; eruption again visible on the hands; skin moist; excoriations behind the ears; the neck and axillæ are more red, not yet moist, eyes more lively, less restlessness; symptoms of ulceration in the mouth. Pergat.

*4th day.* Bowels open only once; eyes not so hollow; excoriation of the nates and neck; continue the emulsion, adding to it half a scruple of argilla (in all two scruples.)

*5th day.* The whole appearance, as also that of the eyes is natural; fontanelle likewise; three yellow evacuations; the soft parts which had been powdered are red; repeat the emulsion only every two hours.

*6th day.* Restless night, again the altered and collapsed appearance; three clayey evacuations; add to the emulsion, Aquæ Cinnamomi, ʒ iij. Extr. Aurant. gr. iv.

*7th day.* Again as on the fifth day, and up to the present time (14th Sept. 1834,) is healthy and thriving.

The two other cases are a complication of the same disease with what the Germans call *Asthma thymicum*, where the child holds its breath when sucking, the nostrils appear stopped up with mucus, and during sleep it suddenly wakes with much dyspnœa and lividity of face. Besides the same treatment as before described, minute doses of cupri sulphas and musk were given. This produced repeated vomiting; the eyes became brighter, the livor of the face disappeared, the cough diminished, respiration was freer, and the child no longer held its breath. We give the formula, as some of our readers may feel inclined to try this plan of treatment in similar cases.

℞ Cupri Sulphatis, Moschi, aa gr. ⅛. Pulv. Acaciæ, gr. ij. Pulv. Glycyrhizæ, gr. iv. M. Pulveres tales, viij. To take one every half hour until repeated vomiting has been produced.—*Hufeland and Osann's Journal, B.* 81. *Juli*, 1835.—*Ib.*

11. *Clinical Observations on the Effects of certain Medicines on the Heart.*—By Lombard, of Geneva.—1. *Assafœtida.* Applied externally in the form of the Emplastrum Galbani comp. (L. P.,) assafœtida seldom fails to quiet palpitations. Internally exhibited, it diminishes and renders more regular the movements of the heart, removes palpitations, and produces a state of tranquility even in those most easily excited.

2. *Camphor.* Given internally, in doses of from three to twelve grains in twenty-four hours, it renders regular the most tumultuous palpitations, and removes the dyspnœa, which often attends hypertrophy of the heart with dilatation. An enlarged heart, with obstructed orifices or dilatation, should be regarded as a muscle fatigued by its constant efforts to maintain an equilibrium between the fluids which enter it and those which are expelled from it; hence the indications are, to give it power by iron and quinine, and to regulate its action by camphor and assafœtida.

3. *Digitalis.* Its sedative action on the contraction of the heart is not constant, but seems to depend on the state of the stomach, mode of life, dose, and mode of administration.

(*a.*) When the stomach is irritated, digitalis often increases the rapidity of the circulation. When given to those whose stomachs are very sensitive, but not in an inflammatory state, it often produces vomiting, and the palpitations and frequency of the pulse disappear; if continued, the pulse becomes much slower, and no inconvenience follows but the shocks from frequently vomiting. The effects of the prolonged use of digitalis on the stomach are not to produce inflammation, but rather a particular state of the gastric plexuses of nerves, analagous to that observed in sea-sickness; and it is best observed by anti-spasmodics and stimulants; such as subnitrate of bismuth, oxide of zinc, then nitrate or acetate of potash, effervescing drinks, ether, and spirits; all of which are employed with advantage in gastralgias accompanied with vomiting.

(*b.*) M. Lombard has very rarely found vomiting produced by digitalis in those who take much exercise, or whose attention is much occupied during its use. This explains why Orfila and others took twenty or twenty-four grains daily, without its diminishing the frequency of the pulse. If they had remained in bed, it would have been otherwise. It has been observed by many that there are very few accidents with digitalis in those cases where the patient is ignorant of the medicine he is taking. Hence patients should be kept in ignorance, and take as much exercise as their condition will permit.

(*c.*) Dose. As a diuretic, it should be very frequently repeated in the twenty-four hours; but, to quiet palpitations, one grain three or four times daily, or three or four spoonsful of an infusion made with one drachm of digitalis to six ounces of liquid, are sufficient.

(*d.*) The peculiar effect of digitalis on the stomach are produced most easily by the infusion; and they are guarded against in some measure only by combining with it ether and aromatics. For the same purpose, magnesia, sub-nitrate of bismuth, sub-carbonate of iron, and oxide of zinc, are combined with the powder. The sub-carbonate of iron is recommended by the Italians, and M. Lombard has found it to be one of the best adjuvants to digitalis; for, by this combination, patients have continued the medicine for many months without inconvenience. It may be given with the infusion. The oxide of zinc is employed by the Germans, and seems to act beneficially by calming an irritable stomach which previously rejected digitalis, not in preventing the symptoms of saturation.

4. *Polygala Senega.* This is a valuable remedy in cases of irregularity of the functions of the heart. Twelve to twenty-four grains of extract, or one drachm of the root infused in four ounces of water, and given daily, appear to diminish the frequency and irregularity of the action of the heart, as well as the consequent sanguineous congestion, in individuals suffering from disease of the heart with dilatation of the cavities.—*Gazette Medicale de Paris*, 10 October, 1835, No. 41.—*Ib.*

12. *Alum in Typhoid Fevers.*—Professor Fouquier, one of the physicians of La Charite, is in the habit of prescribing alum, with considerable success, in certain cases of typhoid fever. When the inflammatory symptoms which generally mark the commencement of fever are succeeded by the symptoms peculiar to typhus, such as weakness of the pulse, fixed and dull expression, diarrhœa, acrid heat of the skin, etc., alum is advantageous. If inflammatory symptoms should re-appear, its use is again counteracted ; so it is if the bowels (which rarely happens in the second stage,) are constipated ; but with these exceptions it may be confidently given, although the most serious nervous symptoms are present. In the stage of collapse, when there is excessive prostration of strength, colliquative diarrhœa, sordes covering the mouth, and fœtid excretions, alum, either alone or with other remedies, acts very beneficially. The diarrhœa diminishes, the tongue becomes moist, and the strength improves. The dose is twenty-four grains daily for three or four days, then increased to half a drachm, and after the same interval to a drachm; when its good effects have been produced, the dose is to be diminished in the same proportion. Gum-water is a suitable vehicle; it may be given in pills, but a solution is preferable.—*Bulletin general de Therapeutique*, November, 1835.—*Ib. for April*, 1836.

13. *On Turpentine in Sciatica.*—By M. Ducros, Jun.—M. Ducros has frequently found sciatica, which had resisted the ordinary means, yield to enemata, containing a large dose of the essential oil of turpentine. He has related several successful cases. In one instance, the pain yielded to one enema, containing an ounce of the oil of turpentine; in another, six lavements were administered in three days, when the neuralgia gave way ; in a third, the quantity was raised to two and a half ounces in each lavement; in a fourth, the lavements were continued for a fortnight. [We know nothing of the practice ourselves.]—*La Lancette Francaise*, No. 110, 15 Sept. 1835.—*Ib.*

14. *On Purulent Metastasis.*—By Dr. R. Froriep.—Dr. Froriep is of opinion that the materials going through the capillaries to the blood are in a state of solution, and do not contain globules; for the porosity of the membrane is not sufficient to permit the passage of globules of pus; so that the appearance of such globules must be coexistent with the effusion of matter. Hence it is incorrect to suppose that a direct discharge of globules of pus takes place into the sac of the pleura, the peritoneum, or the cavity of the arachnoid, as is said, in purulent metastasis; and it is equally erroneous to imagine that when pus ceases to be secreted from the surface of a sore, it has been transported in the current of the circulation, and deposited in some internal organ, where inflammation is going on.

The mechanism of the formation of the so-named purulent metastasis is as follows; if, during the presence of external suppuration, by any contingency, inflammation comes to be kindled up in some internal organ or other, in consequence of the pre-existing affection, it is prevented from displaying the ordinary characteristic train of symptoms; but, attaining imperceptibly its full development, ultimately gains the ascendency of the previously existing suppurative process, in which the discharge is suspended, while the vicarious inflammation, acting upon an organism already enfeebled by disease, pursues a rapid course towards a purulent termination.

In support of this mode of explanation, Dr. F. refers to what has been frequently observed, under such circumstances, on post-mortem examination; particularly to those cases where no deposition of pus is discoverable,—merely a serous exudation, or simply the phenomena of incipient inflammation without the effused products.

In accordance with this view, it is plain that a tonic and stimulent treatment, for the purpose of compelling the pus to flow internally, as is vulgarly said, can only serve to augment the mischief commencing. The measures pursued must be antiphlogistic, proportioned to the strength and constitution of the patient.—*Wochenscrift, f. d. Heilkunde,* No. 8. *Berlin,* 1834.—*Ib.*

15 *On the proper Temperature of Sinapisms.*—The volatile oil, on which the stimulating properties of the powdered seeds of mustard depend, is not disengaged or formed unless water is added to them; but it has been imagined that very hot water was preferable to cold. M. G. Faure, sen., has proved, by many careful experiments, that this is not the case, but that water, when heated to 190° (F.) and upwards, prevents the disengagement of the volatile principle of mustard; consequently, that sinapisms should be made with cold water, and for foot-baths the powdered mustard should be first mixed with some cold water, to which boiling water should be added, to raise it to the necessary temperature. By one of those coincidences which are not uncommon, the same facts have been simultaneously discovered by MM. Geiger and Hesse in Germany. The most satisfactory rationale is, that the sudden heat coagulates the vegetable albumen which forms a coating to each molecule, that prevents the water acting upon it. All causes which coagulate albumen produce the same effect, such as alchohol, strong acids, etc. Cold water, on the contrary, dissolves the vegetable albumen.—*Journal de Pharmacie, Septembre,* 1835.—*Ib.*

16. *Case of Poisoning by Arsenic, successfully treated by the hydrated Tritoxide of Iron.*—By M. Geoffroy.—F., æt. 36, hairdresser, during an attack of delirium tremens, took from his desk a paper which contained arsenic, threw the poison into a glass, poured water on it, stirred it up with his finger, and drank it. Finding that some of the poison remained in the glass, he added more water, and was about to drink it, when one of two persons who were in the room, wishing to see what he was drinking, looked at the paper from which he had taken the powder, and seeing that it was marked arsenic, endeavored to prevent his drinking it, but did not succeed; and he not only swallowed the liquid, but put into his mouth with his finger, the powder which remained in the glass. M. Geoffroy arrived immediately afterwards, and, on discovering the arsenic was contained in the paper, ordered several glasses of sugard water to be given immediately, and procured, as soon as possible, some hydrated tritoxide of iron. In twenty minutes this was obtained, and four or five pints of warm or cold water, charged with it, were given in a quarter of an hour. This produced copious vomiting and a large stool. For the next seven or eight hours this treatment was continued, and the patient vomited and was purged three times. There was neither colic, heat in the throat, nor any symptom of poisoning; he complained of cramps in the fingers, and during the whole time was delirious, talking and gesticulating. The quantity of drink was then diminished; he slept during the night, and in the morning appeared well.

It was ascertained that he had swallowed a drachm and a half at least of white arsenic. From twenty to twenty-five pints of water had been given, in which was suspended the oxide of six ounces five drachms of the sulphate of the tritoxide of iron.

[This is a valuable case, as there was no doubt of the enormous dose of poison taken.]—*Journal de Medicine et de Chirurgie pratiques. Sept.* 1835.—*Ib.*

17. *Hydrated Peroxide of Iron as an Antidote to Arsenic.*—MM. Bineau and Majeste, of Summur, relate the following cases, to prove the efficacy of the hydrated peroxide of iron as a counter-poison to arsenic in the human subject. On the 13th of August last, about two o'clock, five little girls, on leaving school, ate part of a cake containing one fifth of its weight of white arsenic, which had been prepared to kill rats.

L. D., æt. 7, who had eaten a piece weighing about two drachms, had half an hour afterwards, pain in the throat, a sensation of strangulation and vomiting, succeeded by pains in the belly, great thirst, faintness, incessant restlessness, and spasms. Dr. Bineau saw her at four o'clock; she had then vomited five times, and rejected sugared water and milk which she had swallowed. He gave her one grain of tartar emtic, which produced vomiting and two stools. In an hour he had prepared the tritoxide of iron, and by nine o'clock he had given five ounces, in divided doses. During this period she vomited five times, and had one black fœtid stool; there were stupor and slight convulsions of the limbs. At ten o'clock these symptoms were relieved; she slept quietly, and was well the next day.

M. G., æt 5½, swallowed about three drachms of the cake, and a quarter of an hour afterwards vomited. Between this time and five o'clock, she vomited twenty times, and rejected all fluids. After vomiting, faintness and depression, followed by extreme restlessness, pain over the whole body (particularly in the belly and legs,) cold perspirations, livid face, great thirst. At four o'clock there was great and constant stupor, without loss of intelligence. At five o'clock, M. Bineau administered five or six drachms of the hydrated tritoxide of iron, and repeated the dose frequently at first, and gradually increasing the intervals until ten o'clock. During this time she vomited only three times, and the pain ceased; but there was constant and alarming stupor and depression. At ten o'clock the pulse rose; and at four she slept naturally. In the morning she had two stools. No subsequent symptom.

The other three cases were treated by M. Majeste.

Marie B., æt. 7, and Louise, her sister, æt. 5, each ate about two drachms of the cake. At four o'clock, M. Majeste saw them; the face of the eldest was contracted, pale or lived, eyelids injected, great thirst, very hot skin, pulse 120, belly tympanitic and painful, particularly the epigastrium, general depression, and constant vomiting and purging, since the poision had been taken. The symptoms of the younger were rather milder. M. Majeste returned to his dispensary, and in less than an hour prepared twelve ounces of the hydrated tritoxide of iron, and he gave to each two ounces at four doses, in twenty minutes. The vomiting ceased, but returned in an hour, when he gave two ounces more at longer intervals, and a lavement with half an ounce. At eight o'clock the vomiting returned with colic, and an ounce was given to each, with half an ounce in a lavement; the vomiting ceased, and did not return. They passed a good night. With the exception of some little intestinal irritation, and an eruption in the eldest, which yielded to simple treatment, there were no other symptoms.

The other child, aged 9, was less violently affected. The vomiting ceased on taking the antidote, and in eight hours after the attack all danger was over.

*Remarks.* The remedy was prepared in the following manner:—One ounce of iron filings, with four ounces of nitric acid and four ounces of muriatic acid, were introduced into a large glass vessel, and subjected to a gentle heat until the iron was dissolved; to this solution sixteen ounces of cold distilled water were added, and after some minutes the metal was precipitated by introducing two or three ounces of liquid ammonia. The vessel was then filled with common water, agitated, and the whole filtered. This left about twelve ounces of the hydrated tritoxide of iron. A teaspoonful of this weighed about an ounce. The process occupied about an hour, and it was immediately repeated. Its action as an antidote was evident. The dose in each case was very large, above thirty grains of arsenic; and, although vomiting took place, yet a much smaller quantity has been often known to kill, although there was vomiting. The symptoms were always immediately relieved by the iron. The antidote itself appears harmless, as from four to six ounces were given to each child without any ill effects; and, as this is the case, it is advisable, even long after the poison has been taken, to estimate the quantity rather by its effect on the symptoms, than by any proportion to the poison. It is advantageous that this antidote is tasteless.—*Journal des Connaissances Medico-Chirurgicales, November,* 1835.—*Ib.*

18 *Fumigations in Hooping-Cough.*—Dr. Dohm, of Heide, in the duchy of Holstein, has accidentally discovered a remedy for hooping-cough, that promises to be of considerable use in that too-often obstinate and dangerous disease. Two of his own children, a boy and a girl, (the former one, and the latter three, years old,) had been suffering from hooping-cough for between two and three months; during which time several remedies, including belladonna, had been tried in vain. The paroxysms were very frequent and extremely violent, so that the fæces and urine used sometimes to be expelled involuntarily. An accident of this kind occurred one evening during the absence of the father; and, to remove the ill smell thereby occasioned, the bedroom was fumigated, and that to such an extent that the child was enveloped in the smoke. Contrary to the expectation of the doctor, the child had not another attack that night; the cough became much milder, and the repetition of the same treatment soon cured it. This encouraged him to try it in other cases, and he invariably found the paroxysm greatly relieved by it, if not completely stopped. The fumigation was made with the common *species fumales* of the Pharmacop. Slevico-Holst. (Olibani libr. duas, Benzoes, Styr. Calamitæ, sing. libr. dimid., Flor. Lavendul., Rosar. rub., singul. unc. quatuor.) He [we think, very justly,] considers the benzoin to be the most efficient ingredient.—*Plaff's Mittheilungen*, 1*ste Jahgr.*, 1 *und* 2 *Heft.—Ib.*

19. *Treatment of Scald Head*, (*Teigne, Fr. Porrigo.*)—By Baron Alibert.—The hair is cut as close as possible, and the head is washed for some time with a solution of carbonate of soda, or infusion of walnut leaves. An ointment, containing one drachm of soda to an ounce of lard, or two drachms of soda if the hair is thick, is then rubbed in, and the head covered with a piece of blotting paper. Mild bitter infusions are given internally; and, if it is suspected that the parents have a syphilitic taint, antiscorbutic medicines with mild mercurials. If the soda is inactive, it may be replaced by potash. In some cases, an ointment made with the burned leaves of belladonna or stramonium, or the same mixed with water, removes the disease.

[Those affected with scald head in the dispensaries and hospitals of Paris have been put by government under the care of two brothers, named Mahon, who have cured above 50,000 cases in these public establishments. They begin by cutting the hair short, (to within two inches of the scalp,) and, after removing the scabs with emollient poultices, they wash the head with soap and water for several days, until it is very clean. They then apply a depilatory ointment, which removes the hair slowly and without pain, and sprinkle the head with a powder which forms the basis of the ointment, taking care to keep the scalp very clean. The composition of this ointment they keep secret. M. Rayer has endeavored to supply its place with one consisting of an ounce of prepared chalk, two drachms of subcarbonate of potash, and one drachm of powdered carbon, mixed with lard; the quantity of which is to be increased or diminished in proportion as the scalp is more or less inflamed. M. Biett often prescribes the following lotion instead of the depilatory powder:

℞. Sodæ Sulphur. iij.; Saponis Castil. ℥ss.; Alcohol, ʒij.; Aquæ Calcis. lb j.

Misce, fiat lotio.

M. Giscard, an army surgeon, employs successfully this ointment:

℞. Axungiæ, lb. ij.; Sulphuris, ℥ij.; Pulv. Carbonis, ℥viij. M. fiat ung.

After shaving the head, he directs a layer of this ointment to be applied over it, to be washed off two or three days afterwards with a solution of black soap. This application, repeated five or six times, has cured the most complicated cases.]—*Journal de Medecine et de Chirurgie pratiques, November,* 1835.—*Ib.*

20. *New Mode of Treating Luxations of the Sternal End of the Clavicle.*—By M. Velpeau.—No luxation is more easily reduced, or with more difficulty retained in its place. The difficulty is owing to the endeavor of the surgeon to oppose the power which tends to carry the clavicle forwards, instead of relaxing the muscles whose action oppose his wishes. Anatomy and experience show that in these cases the anterior sterno-clavicular and inter-clavicular ligaments are broken, and that dislocation necessarily follows, as the articular surfaces are so constructed that they do not offer any resistance to the muscles, which immediately displace the end of the bone: the pectoralis major and internal portion of the deltoid drawing it forward, the trapezius and sterno-cleido-mastoideus drawing it upwards; whilst both sets are assisted by the large muscles of the trunk attached to the scapulæ. The deltoid, pectoralis major, sterno-cleido-mastoideus, and trapezius are relaxed, and the rest neutralized, by carrying the elbow inwards and forwards to the lower part of the sternum, so that the hand may rest upon the opposite shoulder; when this is done, the bone returns to its place. It can be retained there by the following apparatus: A towel, folded thrice, is first placed round the thorax, and retained by braces attached to its upper border. The arm is then placed in the necessary position, and held by an assistant. The surgeon fixes the end of a bandage (eight yards in length) under the armpit of the sound side; brings it behind over the luxated clavicle; then downwards over the part of the arm; passes it behind and beneath the elbow; then again under the sound armpit, behind the chest, above and before the injured clavicle, so as to make three or four turns like the first. Next he passes the bandage, which has just embraced the elbow, over the fore-arm, then on the sound clavicle, between the hand and the neck, instead of passing it under the armpit as before; after which he passes it downwards behind the thorax, towards the elbow, bringing it again on the fore-arm and clavicle, and making thus three or four diagonal turns. After this the bandage is not again passed under the elbow, but is passed two or three times below upwards round the chest and the bent arm; the remaining part is expended by making a few turns like the first, fixed by two or three circular ones. The whole is fixed by pins, and secured by a napkin covering all. This bandage may remain a month or more without any displacement. A more simple contrivance is a belt, or band, passing round the body, and having in front a deep pouch for the elbow. When the belt is fixed, and the elbow placed in the pouch, the belt must be drawn forcibly upwards towards the clavicles, and fixed there by braces over the shoulders, which are attached to the upper border of the pouch, as well as to the edges of the body-belt.—*Journal Hebdomadaire de Medecine, Mai* 30, 1835.—*Ib.*

21. *Medical Jurisprudence and Toxicology.—Experiments on Peroxide of the Hydrate of Iron as a Counter-poison to Arsenic. First Series.*—By MM. Miguel and Soubeiran, of Paris.—The French chemists have not been tardy in endeavoring to test the efficacy of this antidote to arsenic, which was discovered by M. Bunzen.—The following series of experiments were undertaken subsequently to those of M. Leseur, which were communicated to the Academy by Orfila, who considered that the German chemist, had not exaggerated the importance of his antidote. The French experimenters found that if a large dose of arsenic was given to dogs and they were allowed to vomit, it produced no effect: it was necessary therefore to tie the œsophagus of those dogs on whom any experiments were to be made. As tying the œsophagus is a fatal operation, it was essential to ascertain the length of time that a dog would live after this alone was performed; and also the time which arsenic alone took to produce death when the œsophagus was tied after its introduction. A dog whose œsophagus was tied simply, died in seventy-eight hours: two other dogs, to whom nine and twelve grains of arsenic were given, and who were prevented from vomiting by tying the œsophagus, died in two hours and two hours and a half. In all the following experiments, recently prepared peroxide of hydrated iron mixed with water was used, in the proportion of twelve parts to one of white arsenic.

Twelve grains of arsenious acid mixed with water was given to a little dog, and immediately afterwards the iron: the œsophagus was then tied; for some time he endeavored to vomit, but two or three hours afterwards all symptoms had left; twenty-four hours after the operation the ligature was removed; fluids were given, but deglutition of solids was impossible, and the dog died on the sixth day. Two large dogs were similarly treated with a larger dose of arsenic (eighteen grains :) the ligature was not removed. One lived seventy-eight, the other eighty-four hours.

The salutary effects of the hydrate of iron cannot be doubted in these three cases; for from the previous experiment, the arsenic alone would have killed them in two or three hours, whereas they lived as long as they would have done had the ligature only been employed. Another series of experiments was performed to ascertain if the iron would arrest the effects of the poison after they had lasted some time. The poison was shown, by a previous experiment, to kill in two hours and a half: and this fact was confirmed by giving a dog eight grains of arsenic, after his œsophagus had been tied, and introducing the antidote two hours and a half afterwards: he died a quarter of an hour after this injection. To determine how long after the poison was taken the counter poison was useful, four experiments were made. Eighteen grains of arsenic were give to a middle-sized dog, the œsophagus was tied, and presently the efforts to vomit were very violent; an hour afterwards an opening into the œsophagus was made below the ligature, the iron was injected, and a second ligature applied. The animal lived ninety hours after the poison had been given. Two spaniels were treated in the same way, the antidote being injected one hour after the poison; eight grains of arsenic was given to one, who lived ninety hours; and twelve grains to the other, who lived thirty-eight hours. Eighteen grains were given to a large dog and the iron introduced *two* hours afterwards: he lived thirty hours.

In all four cases death was greatly delayed by the iron, and in the first experiment on a very strong dog, the poison was neutralized, as he lived as long as if a ligature only had been applied. The conclusion must be admitted, that the iron acted as a counter-poison for some time after the arsenic had been swallowed. The unfavorable circumstances under which the antitode was given, strengthen this conclusion; the animals had undergone an operation which always produces on them great depression; the whole of the arsenic had been retained in the stomach for an hour or two, and nothing had been given to diminish its local or constitutional effects. In two experiments it was found that when arsenic was mixed with the fatty matters, the iron was less efficacious as an antidote; probably from the arsenic being covered by the fat, and protected from the action of the iron.

The hydrated peroxide of iron used in these experiments was procured in the following way. Sulphate of iron of commerce was mixed with five or six times its weight of water in a platinum or porcelain capsule, and when it boiled, small quantities of nitric acid were added until the ruddy vapor ceased to ascend: this was to bring the oxide of iron to its maximum of oxygenation. The liquor was then diluted, and the iron precipitated by liquid ammonia. The precipitate was washed and mixed with a small quantity of water until it had the consistence of clear "bouillie." As it cannot be weighed in this state, thirty-six times as much sulphate of iron is required as the poison taken, for the sulphate contains one-third of its weight of peroxide. It should be kept as a hydrate, for the dried powder does not act as an antidote. The chemical change consists in a conversion of the arsenious acid into arsenite of iron. Three times the quantity of the hydrated peroxide of iron is sufficient to neutralize arsenious acid in solution, and the decomposition is instantaneous; but as the arsenic when used as a poison is almost always swallowed as a powder, a much larger proportion of the antidote is advisable, for the action goes on very slowly. Even then however it must neutralize all ill effects, if it is in contact with the arsenic, for as soon as the smallest quantity of the poison is dissolved, the oxide of iron acts upon it and precipitates it.

In conclusion it is recommended in all cases of poisoning by arsenic to give the patient large quantities of hydrate of the peroxide of iron; at the same time also to encourage vomiting, to get rid of undissolved arsenious acid from the stomach; and to repeat the hydrate as long as there are symptoms of any of the poison remaining.—*Bulletin General de Therapeutique, December*, 1834.—*Ib.*

22. *Ulcer of the Pylorus.*—By Benjamin Wilscn.—On Wednesday the 6th of August about 7 o'clock in the evening, I was requested to visit Mr. M——— as soon as possible, he having been taken seriously unwell.

Upon enquiry it appeared that he had not felt well for several days, and complained of a peculiar sinking sensation in the stomach, as if (as he expressed it) his stomach was quite gone, and sometimes he felt as though a heavy weight was pressing upon it; that when walking in the verandah he was seized with a sudden severe pain accompanied with a sensation as if something had burst, or given way in or near his stomach, at which time it appears he fell down. He said he was convinced that an abscess of the liver had burst interally and that he should never survive it. He was in a cold sweat, pulse very small and feeble, countenance pale and anxious, with great restlessness, a dreadful weight at his stomach, with an inclination, to vomit, but unable to get any thing up, had taken a glassful of warm water, to incourage it. Boiling water being at hand, hot fomentations were immediately applied to the region of the liver and the stomach, and I sent off for a supply of leeches, the feeble state of his pulse, and nearly exhausted state of his system generally, precluded my taking blood from the arm; about a quarter of an hour after my arrival he vomited twice: each time about ½ a pint, of dark brown fluid as if tinged with blood, and flakes of what appeared thick matter or pus floating in it, which led me to suppose that Mr. M's. opinion might be correct as to the bursting of an abscess of the liver. After this his stomach appeared more settled, and I gave him 10 grs. of calomel and a draught of camphor mixture containing 45 drops of laudanum; the leeches also arriving at this time 40 were applied to the hepatic region, the seat of pain. Ten o'clock. After the leeches had been applied a short time he complained of feeling very weak and faint, and as his pulse had become more feeble, I was under the necessity of removing them immediately and staying the bleeding. From this time to 12 o'clock no favorable change took place, for though he said he felt the pain less severe, still the anxiety and restlessness continued; requesting occasionally to have a cup of warm tea, or a glass of camphor mixture. About half past 12, I repeated the 10 grs. of calomel and a draught with 35 drops of laudanum; after taking which he said he should like to endeavor to get a little sleep; he continued to doze and had some disturbed sleep, and about half past 2 o'clock he awoke and expressed a wish to sit up in bed, which he did for about 5 minutes, when he became very restless, saying he felt himself getting worse and felt assured that he should not live till day-light. As his bowels did not appear at all affected by the calomel, an ounce of castor oil was given, which he retained though he was afraid it would make him sick. He continued to doze, occasionally asking for a little warm tea, till day-break; and as the castor oil did not appear to be acting upon the bowels, I went about 5 o'clock to the hospital for an enema syringe and to prepare a purgative mixture, pills, and a blister; the mixture of epsom salts and infusion of senna I immediately dispatched with directions for a large wine glassful to be taken every hour till the bowels were purged. During my absence he felt himself worse and directed Mr. Lang to send for me, he took a dose of the aperient medicine immediately on its arrival, and on my return he appeared in great distress that the medicine did not appear to act on his bowels, and was convinced that something serious had happened to his stomach, as his bowels were always very easily moved, he took another dose of the mixture and washed it down with some tea, he also expressed a wish occasionally to have a little soda water which he was allowed to take during the night; said that it was all useless, as what he took, he felt go down to the bottom of his belly, and it did not appear to enter his bowels; two purgative enemas were afterwards administered but without success, and they were returned without bringing any thing away; from this time (9 o'clock,) he became gradually worse, and expressed his conviction that he was in a dying state. He refused to take any more medicine, and also some warm wine and water which was recommended and had been frequently during the night, he being in a cold sweat and his pulse scarcely perceptible; he continued to become weaker from 9 o'clock to 11, with occasional fits of hickup with cold sweats, very restless and countenance extremely anxious. About 11 o'clock his breathing became stertorous, with frequent deep sighing and hickup, pulse scarcely perceptible at the wrist, from 12 o'clock he continued to sink very fast, becoming comatose, the eyes closed, breathing short and stertorous, and died about one-fourth to 1 o'clock, P. M.

*Post Mortem Examination 24 hours after death.*—By Benjamin Wilson.—Abdomen in a state of great inflation, and on perforating the peritoneum, a strong gust of highly offensive air was expelled. On laying open the peritoneum along the whole length, a large quantity of dark brown fluid, (say two pints) appeared diffused among the intestines, which upon being collected and examined appeared to be the contents of the stomach, with the castor oil floating on the surface. The stomach still continned in a state of great inflation; however on carefully drawing out the pyloric end from under the left lobe of the liver, another gust of fetid air burst out of a small orifice discovered on the outer front surface of the pylorus from which some fluid still remaining in the stomach continued to escape; on minute examination, the coats of the pylorus at its junction with the duodenum to the extent of about two inches were very much thickened and the passage so contracted as to be rendered nearly impervious;—the ulcer was situated just at the edge or commencement of the contraction, on the front outer surface of the pylorus. On turning up and examining the under concave surface of the left lobe of the liver, and part in contact with and resting upon the pylorus quite livid and in a sphacelous state. The ulcer was of an oval shape with regular round thickened edges and was large enough to admit easily the little finger: on pressing the stomach, the air and a little fluid which still remained, continued to escape from the ulcer. No disease appeared to exist in any other part of the stomach. The right or large lobe of the liver was reduced in size, and much smaller than natural, the substance was of a dark blue slate color, with great congestion; there were no adhesions, though nearly the whole convex outer surface from the lower thin edge upwards, was covered with a deposition, elevated above the surface, apperently ulceration, the effect of long continued chronic inflammation; the substance of the right lobe generally, was in a state of great disease. The left lobe had a very different appearance to the right, being of a yellow or buff color on the outer convex surface, and appeared much larger than natural, the under concave surface had assumed a dark lived color and appeared in a sphacelous state. The gall bladder was nearly filled with pale yellow bile. The Omentum, and whole surface of the peritoneum lining the abdomen in a state of high inflammation, and the vessels of the former gorged with blood, the outer peritoneal coat of the intestines in a state of general inflammation; the bowels were inflated with air, but otherwise quite empty, the lungs appeared in a perfectly healthy state.

It appeared that Mr. M. had frequently complained of a peculiar sinking sensation in the stomach, from which he had suffered previous to the last fatal attack; during the last three days he felt he said as if his stomach were quite gone and occasionally as though a heavy weight was pressing upon it. I am of opinion that inflammation had been going on for a long period of time, and that during the process, adhesion had formed between the under concave surface of the left lobe of the liver and that part of the pylorus, on which the ulcer was situated, which parts would naturally be in contact. On the night of the attack these adhesions giving way during the act of falling, or the effect of vomiting, the contents of the stomach made their escape into the abdomen, for, as he expressed it, he felt as if something had given way in his stomach; and was convinced that an abscess of the liver had burst internally, that some fatal occurrence had taken place, and that he should never survive it. He mentioned having taken occasionally blue pill over night, with a purgative the following morning, which with starvation for a day or two he said generally relieved him; he had taken no solid food during two or three days, and from the appearance of the ulcer which I think had existed some time, I am of opinion that had he taken a full meal the fatal occurrence might have taken place earlier, the adhesion to the under surface of the liver having alone been the means of preventing it. From the exhausted state in which I found Mr. M., on first seeing him, in a cold sweat with scarcely any pulse at the wrist, and a countenance of extreme anxiety and restlessness with an insupportable weight and oppression at the stomach, exclaiming "what can have happened? I never felt such a sensation in my life, I am sure I shall die." I was led to fear that some serious injury had occurred and the very unfavorable change which subsequently took place convinced me that nature had suffered a fatal and irrecoverable shock, and the circumstance of finding the contents of the stomach, the senna mixture and castor oil, diffused among the intestines in the abdomen, evinces that any measure adopted for the relief of the patient in the present melancholy case must have proved alike unavailing.—*India Journal of Med. Science.*

23. *Singular Case of Lead Imbedded in the Tibia.*—By JAMES ATKINSON.—Whilst I was attached to the General Hospital in Calcutta, I had occasion to amputate the leg of an old soldier, in consequence of a gangrenous spreading ulcer just above the ancle. When the operation was finished we proceeded to dissect the limb in order to examine the internal condition of the diseased parts, and on extending the incision along the tibia, an irregular line of lead was discovered imbedded in the bone, with the external surface smooth. This was several inches above the ulcer, with which it had no connection whatever, the bone in the intermediate space being quite sound. Upon being questioned on the subject the man recollected that he had received a wound in action, on the shin, many years before, but had suffered no inconvenience from it, as the slight laceration had speedily healed up, and had left no pain or scar to mark the place, or recall the circumstance to his mind. The quantity of lead appeared to be about as much as might be contained in a musket-ball, scattered and diffused as seen in the annexed outline.*

*The plate shows an irregular mass of lead extending two or three inches in length along the tibia above its centre.—JUN. ED. MED. JOUR.

But how so singular a result could have been produced it is difficult to imagine, a ball striking the bone would in ordinary cases fracture it, but the resistance in the present instance seems to have been as great as if the lead had struck a dead wall. And it is equally difficult to conjecture how the lead could have acquired its elongated and irregular form. The bony bed in which it lay, was not in any degree exfoliated or diseased, indeed the lead had, as it were, become a part of the man, for the periosteum and skin had closed over it in perfect harmony, leaving no indication of the presence of a foreign body.—*Ib. March,* 1835.

24. *Elephantiasis of the Arabians.*—By LISFRANC.—Maria Provots, aged 31 years, who had been employed in laborious occupations, was admitted into the hospital of La Pitie. She had been afflicted since seven years of age with the Arabian elphantiasis of the right leg, which was amputated on the 29th of March 1823. Her health remained pretty good for five months, after which the disease appeared in the hand and forearm, and inferior half of the right arm. When the joints of the arm were moved, which could only be done in a very contracted manner, a distinct crepitation was heard in the elbow, wrist and finger joints. By the alternate application of leeches and blisters, with an infusion of hops and anti-scorbutic wine, the cure was nearly completed, when the patient insisted on leaving the hospital. Three hundred and fifty leeches were applied in numbers of from twenty to thirty at a time, and forty-seven temporary blisters were used for the cure; and as long as the arm remained inflamed, it was covered with emollient poultices, to which laudanum was added when the pain was acute.

In January 1825, the articulation of the elbow, wrist, and hand of the right side, particularly above the course of the lymphatic vessels, became suddenly painful; a knotted, and enlarged cord, resembling a string of sub-cutaneous tubercles formed in the course of the pained parts; which was indicated by a red streak. The pain increased and extended to the axilla where the lymphatic glands were enlarged; and the erysipelatous redness covered the lymphatics and extended over the right arm, with the exception of its superior part. The irritation extended to the subcutaneous cellular tissue followed by considerable swelling, and it was so tender that the simple contact of the clothes was distressing to the patient. The elbow and wrist and joints with those of the hand were stiff and contracted; at the same time she was tormented with an insatiable thirst; long and severe shivering fits, anxiety, acute fever, severe headache, and delirium. There was no vomiting. Great heat succeeded the shivering, followed by general and abundant perspirations, particularly in the affected arm, with a diminution of the febrile symptoms.

The general symptoms diminished, and again increased in the form of an accession, at the interval of four hours, or, to speak more correctly, the fever with the exacerbations, which preceded a redness more intense, and pain more acute. The accessions commenced by coldness, sometimes confined to the affected parts, and by more intense thirst. The great heat was succeeded by very abundant perspirations, sometimes general, at other times local. Often the pain, shivering, and perspiration existed at the same time, and were confined to the diseased member, the swelling and hardness of which increased gradually, while the pain and heat diminished a little during the intermission. The catamenia were suppressed, followed by severe headaches. Lumbago appeared, which with the other symptoms were removed by venesection.

She was again admitted into hospital on the 31st March 1825, with the whole arm, with the exception of its superior half, quadruple its natural size, red, very hard, uniform and cylindrical. It felt very hot, and tender, and very painful, particularly at the stiff contracted elbow and wrist joints, and joints of the hand. The system was powerfully affected during the attack, and the frequent and irregular accession was indicated by coldness, which was generally confined to the member affected. The thirst, heat, and abundant perspiration succeeded or accompanied these phenomena, which considerably diminished in the intervals of the accession, with the exception of the tumefaction, the hardness of the member, and the difficulty of its being moved increased with each attack.

M. Lisfranc prescribed repose and the horizontal position of the member; and when the pain was acute, emollients with the addition of opiate preparations, as laudanum, with a warm and equable temperature. Antiphlogistic regimen was enjoined with mucilaginous and acid drinks. The five first days they applied three times, sixty-five leeches, without much advantage. The other remedies were continued. The redness, swelling and hardness of the member continued, and at an interval of two or three days one or two temporary blisters were applied. When they produced too severe inflammation, poultices and tepid baths were used. The discharge of serum which they occasioned was very abundant, so as to oblige the arm being dressed twice a day. At the end of a month the blisters had diminished the size of the arm. Forty or fifty small scarifications (mouchetures) were made, which caused little bleeding, but an abundant flow of serum which was promoted by local warm baths. The first discharge (from the patient's account) continued eleven days, during which the scarifications were twice repeated, and the arm was much diminished in size. The blisters and scarifications were alternately used, and from time to time an emollient poultice. Two or three days after the scarifications, one, two, or three blisters were applied.

On the 1st of May the pain was slight, the swelling and hardness diminished; the scarifications were repeated every five or six days, followed by the immediate immersion of the arm into warm water, and a considerable quantity of serum continued to be discharged. On the 15th of May, the hand was nearly reduced to the natural size: the forearm, of which the volume was much diminished, was softer, and the movements of the finger and elbow joints were pretty free; while the wrist joint was still rigid, and the arm appeared of its natural size. The scarifications were now principally applied on the upper part of the forearm. The arm continued gradually to improve until the 11th June, when severe headache with redness of the right eye occurred; purgatives, mustard baths, emollient applications to the eye were used; veal tea was added to the low diet that had been allowed. On the 15th of June the symptoms increased in intensity for which she was bled; two days after the symptoms had disappeared. The scarifications were continued, particulaly where there was most swelling. It was particularly the day after the scarifications had been made that a diminution of the swelling and hardness appeared. The arm slowly returned to its natural form. Since the cure the woman has been discharged several weeks.—*Ib. July*, 1835.

25. *Remarkable Monstrosity.*—M. ANGELO BOSSINI relates the following case in the *Annali Universali di Medicina.*

During the night of the 15th February, 1834, he was called to attend a woman of the name of Francesco, 43 years of age, the mother of several healthy and well-formed children. She had been many hours in labor, and the midwife in attendance considered the case to be difficult. He found two feet already protruded, one of which, according to the midwife, came down immediately after the rapture of the membranes, the other she had brought down. M. Bossini then sought for the arms, and having ascertained the situation of the umbilical cord, he brought one, as he thought, to the vulva, when he found it was another foot. He, of course, considered now that he had to do with a twin case, but soon discovered that this third limb belonged to the same trunk as the two preceding. By carrying his manœuvres a little further, he found the fourth limb, which he brought down, and the hips were then tangible, when he ascertained that the four lower extremities with a portion of two trunks were united into one at the umbilicus. The narrator of the case then gave the body the motion of semi-rotation, by which he was enabled to reach the arms, and bring them down. By pursuing the manœuvres of rotation, and depression, the monster was born. It presented two heads, two necks, uniting into one large thorax, with two arms, one of which appeared to belong to each fœtus. There were two spinal columns, two scapulæ, two ani, and two sets of the genital apparatus (female.) There was only one placenta. The extraordinary production died during the progress of labor. The right fœtus was more developed than the left, but they both appeared to be of full growth.—*Ib.*

26. *On the use and abuse of cold applications.* BY ROBERT J. GRAVES, M. D.—In affections of the head occurring in acute diseases, and attended with raving and loss of rest, it is a very usual practice to direct the application of cold lotions to the shaved scalp.

Permit me, gentlemen, to make a few remarks upon this important subject. I wish I could make myself well understood on this point, for I have seldom met with any person who seemed to bear in mind the true principle upon which cold is applied as a means of repressing local heat. In cases of determination of blood to the head occurring in fever, the common practice is to have the head shaved and cold lotions applied. Enter the room of a patient who is using cold applications, and you will observe the process conducted with great apparent nicety; the head is accurately shaved and carefully covered with folds of linen wet with a lotion to which spirit of rosemary or some odoriferous tincture has communicated an agreeable and refreshing smell; but when you come to examine the patient, you find his head smoking and the heat of his scalp increased. The nurse applies the lotion once every half hour, or perhaps not so often; indeed, she seldom repeats the application until her notice is attracted by the steam rising from the patient's head, or until she herself, awaking from a comfortable sleep, and going over to examine the state of the patient's head, finds the folds of linen which cover it as hot and dry as if they had been hung before a fire. Whether applied to reduce local inflammation in any part of the body, or to cool the scalp in determination to the head, cold lotions as ordinary employed do infinitely more harm than good. The cold is applied at distant intervals, its effect soon ceases, and reaction constantly takes place, leaving the part as hot or even hotter than it was before.

If you put your hand into snow for a few moments, and then take it out, it quickly resumes its natural heat; and if you repeat this at considerable intervals, so as to give time for reaction to occur, the vessels assume a more energetic action, and it becomes hot and burning. If you continue to keep it in the snow for a long time, its heat becomes completely exhausted, reaction does not take place until after a considerable period, and very slowly, and the hand remains at a very low temperature for a good while. Bear this in mind, for it will direct you in the application of cold to reduce local heat. If cold applications be used at such intervals as to allow the scalp to react and resume its heat, rely upon it, it is much better to forbid them altogether. Where you wish to apply cold with effect, let it be done by relays of folded linen, wet with any frigorific mixture, and repeatedly applied to the scalp so as to leave no smoking, or what is much better, get three or four bladders, put into each a quantity of pounded ice, and apply one over the crown of the head, one on each side, and lay one on the pillow for the back of the head to rest on.

There is a vast difference between a thing being done and its being well done, so it is with regard to cold lotions; so difficult is it to ensure their proper application, that I have entirely given them up in hospital practice, and rarely order them in private. I have been induced to abandon them in consequence of witnessing so many instances in which my directions were neglected, and consequently the cerebral congestion was augmented by their mal-application. Another serious inconvenience frequently arises from their use when applied in a slovenly manner, which is the danger of cold arising from the pillow and bed clothes being wetted.

It is a curious fact that the head is the only one of the three cavities with respect to which long established custom has laid down the maxim, that when its contents are inflamed we may cool the surface over it, while, in inflammatory affections of the thoracic or abdominal viscera, this practice is avoided as dangerous and inapplicable. Latterly, however, some medical men have been inclined to question the grounds on which cold applications have been rejected in the two latter cases, and some have even declared that they have used ice poultices in inflammation of the chest and belly with great success and perfect safety. I am not as yet prepared to adopt this practice, although I must confess that a review of the subject might incline me to give up my prejudices on this point. It is certainly but reasonable to think that what is true of the one may be also true of the other, and that the application of cold to the head and heat to the chest and belly has nothing in its favor beyond mere custom. It should be recollected, however, that the head and face are more accustomed to cold than the chest and belly, and hence are less liable to any mischief likely to arise from its application in an intense degree. Still I am inclined to think that there is much prejudice connected with the practice of applying cold to the head; and I have very little doubt that if the matter was properly investigated, and a number of experiments made, it would lead to the abandonment of cold applications in most inflammatory diseases of the brain. In fevers, I can say positively that in the majority of cases they are positively injurious, *as usually applied;* sponging the bare scalp with tepid or warm vinegar or water, or *even frequently repeated steeping of the head and temples* will often succeed much better in abating the head-ache and restlessness of fever than any cold applications whatsoever. In 1832, a violent influenza, accompanied by most distressing head-ache, attacked thousands in Dublin; this intense pain in the head was relieved by nothing so effectually as by diligent steeping of the temples, forehead, occiput, and nape of the neck *with water as hot as could be borne.*

I do not speak here of the application of cold to the head, for the purpose of relieving local heat and inflammation, but to produce an effect on the whole system. Cold thus applied is of decided and unequivocal value. You are aware that in cases of fever accompanied by symptoms of high mental excitement and great heat of skin, the use of cold dashing has produced the most extraordinary effects. Again, if a patient has taken too large a dose of prussic acid or any other narcotic, the best mode of rousing him is by pouring water on his face or chest from a height. In Turkey, if a person happens to fall asleep in the neighborhood of a poppy field, and the wind blows over it towards him, he becomes gradually narcotised, and would die, if the country people who are well acquainted with this circumstance did not bring him to the next well or stream, and empty pitcher after pitcher on his face and body. This occurred to my friend, Dr. Oppenheim, during his residence in Turkey, and he owes his life to this simple but effectual treatment.—*Lond. Med. and Surg. Journ. 21st March*, 1835.—*Amer. Jour. of Med. Sciences.*

27. *Recovery from Asphyxia in a new born infant.*—The following case, very remarkable and interesting from the length of time animation was suspended, has been communicated to the Edinburgh Medico-Chirurgical Society, by Dr. Macwhirter; and is contained in our esteemed *Edinburgh* contemporary for January, 1835.

"I was called, at about half past 11, P. M., to a lady who had been several hours in labor. I found the *os tincæ* expanded to the extent of a crown piece; the membranes pressed forward by every pain, and the presentation '*breech*,' pain recurring regularly and forcibly every four minutes. About a quarter before one, the membranes gave way, the *liquor amnii* was discharged, and the labor, which was a first one, advanced slowly, until half past one, when the breech was born. Suspecting that resuscitation would be necessary, I desired the nurse to have warm water at hand. The body and head were long *in transitu;* the funis was round the neck twice; I disengaged it, but could feel no pulsation. I got my fingers into the mouth of the fœtus, and succeeded in bringing it beyond the verge of the perineum, but it did not breathe. In about ten minutes, by management and the uterine action, the head was delivered, and along with it the placenta.

"The infant appeared *dead;* indeed, it was thrice felt convulsed *in transitu.* The face was as white as paper, but there was some color in the lips; still no pulsation of the heart could be felt.

"I placed the child, with the placenta attached, in a warm bath; gently inflated its lungs with my own breath, in the usual way; rubbed brandy on the chest, abdomen, and head, spine, extremities, &c. As the funis when cut did not bleed, I therefore tied it. After immersion for about half an hour, I took it out of the bath, dried, and wrapped it in warm flannel, and made the nurse carry it near the fire. I then continued the gentle inflation from time to time, and the spirituous friction, to the extent of nearly two bottles of brandy and whiskey, occasionally slapping the bottom, when at length, about forty or fifty minutes after birth, it gave a sob. This was indicative of existing life, and encouraged me to persevere. It continued to sob or gasp at intervals of a minute or two, and I now found that I could feel the heart beat.

"The above means were most perseveringly continued, until the circulation and breathing very gradually increased. At the end of an hour and a half, the child gave a whimper; the eyes opened; the lips became red; it breathed regularly; cried lustily. It lived, and continues to live."—*Ibid.*

28. *On Sleeplessness, and its Treatment.* By R. J. Graves, M. D. (Extracted from a clinical lecture delivered at Meath Hospital, Dublin.) Sleeplessness is a very curious result of disease. It accompanies certain morbid conditions of the system brought on by actual disease, or by grief, care, and various other forms of mental disturbance, continues to harass the unhappy sufferer night after night, and frequently resists the most powerful and decided narcotics. I do not intend to enter into any inquiries respecting the different states of the constitution in which it occurs; my purpose is merely to offer a few practical remarks on the more obvious and striking examples, with the view of illustrating the cases to which I have directed your attention.

There is a form of sleeplessness which is frequently the precursor of insanity, and which has been well described by my friend Dr. Adair Crawford. The watchfulness in such cases is accompanied by the well known symptoms of incipient mental derangement, and its treatment is therefore inseparably connected with that usually resorted to in cases of threatened insanity, and embraces the employment of means moral as well as physical. Of these it is not my intention to speak; I may observe, however, that Dr. Crawford has found opium, gradually increased to very large and frequently repeated doses so as to produce sleep, the best remedy.

In the case of jaundice, the patient passed several nights without any sleep. He was just beginning to recover from the jaundice when this new symptom appeared, and I directed your attention particularly to the circumstance, because every manifestation of nervous derangement connected with jaundice should be carefully watched. It frequently happens that jaundiced patients sleep too much, and in some cases the disease is accompanied by convulsions, succeeded by coma, most alarming symptoms, and almost invariably the harbinger of a fatal termination. Dr. Marsh was the first who directed our attention to the great fatality of those cases of jaundice in which convulsions occur: I have seen but one instance of recovery. It was in the case of a gentleman laboring under icterus, very considerable hepatitis, with enlargement of the liver and anasarca, with ascites. He was treated by Dr. Osborne and myself, and had at least a dozen long and violent convulsive paroxysms, ending in coma, succeeded by temporary forgetfulness and fatuity. Repeated leeching of the right hypochondrium, active purgation, and mercurialization of the system removed all the symptoms of disease, and he slowly but perfectly recovered. A very able and original writer, Dr. Griffin of Limerick, has detailed the particulars of some interesting cases of this nature in the *Dublin Medical Journal.* You perceive, therefore, that in jaundice every thing denoting an unusual state of the nervous system, whether it be too much sleep or too little, demands your attention.

In this man's case, the jaundice was the result of an attack of hepatitis. We treated it with leeches, blisters, and the use of mercury, and in the course of a few days the stools became copiously tinged with bile, and symptoms of improving health appeared. At this stage, the dejections being bilious, but the jaundice still remaining, he began to exhibit symptoms of restlessness and nervous irritability, and finally became perfectly sleepless. Here, gentlemen, we had to deal with a new sympton, extremely harassing to the patient, and likely to react unfavorably on the original disease. As a preliminary step I determined to evacuate the bowels, and for this purpose I prescribed a purgative draught, consisting of five ounces of infusion of senna, half an ounce of sulphate of magnesia, a drachm of tincture of senna, and a scruple of electuary of scammony. My object was to purge briskly, and then give a full narcotic. In all cases of jaundice depending on hepatic derangement, after you have succeeded in producing bilious evacuations, you should never omit prescribing an active aperient every second or third day for the space of ten days or a fortnight, with the view of carrying off the remains of the disease, so as to prevent the occurrence of a relapse. Hence you will find such cases very much improved by the use of Cheltenham water, taken every day for three or four weeks *after the reappearance of a bilious tinge in the alvine discharges.* The stimulus of the purgative causes an increased flow of bile into the intestines, which removes the hepatic congestion, and carries off what is popularly termed the dregs of the disease, and promotes a rapid and complete recovery. It is a simple but successful practice, and I would advise you never to omit its employment in cases of this description.

With respect to purgative mixtures, I may observe that you should prescribe a larger quantity of the infusion of senna than is generally ordered, if you wish to secure its certain and decided operation on the intestines. Hospital nurses, who reason from facts and experience, know this, and when directed to give a senna draught they always give a small tea-cupful. They administer from four to six ounces at a time, and I have observed that in this way the action of the medicine is more certain, and the benefit derived from it more extensive. I am convinced that the usual mode of giving this valuable purgative in private practice is bad; the quantity given is too small, and consequently it is necessary to repeat the dose several times, a mode of proceeding apt to occasion much nausea and griping, I would therefore recommend a quantity varying from three to six ounces, to be administered in all cases where the patient's condition will admit of free purging. A most accurate observer of the effects of medicines, Mr. Kirby, is in the habit of ordering purgative mixtures in chronic cases to be taken at bed-time, and not, as is usually done in the morning. He asserts that their action is milder and less irritating to the bowels when the patient lies in bed and is asleep until the period of their operation, than if he were up and about.

After the purgative had produced four copious discharges, I prescribed eight minims of black drop, to be taken at a late hour in the evening. Whenever I give opiates to procure sleep, I always observe the rule laid down by Dr. M'Bride, (a celebrated physician of this city,) to select the period at which nature usually brings on sleep, and which varies according to circumstances and the habits of the patient. Whenever you have to deal with watchfulness in patients laboring under morbid states of the constitution, as, for instance, hectic, inquire when the tendency to sleep usually occurs, and administer your narcotic about an hour or two before its occurrence. It is between three and five o'clock in the morning that the inclination to sleep is strongest, it is about this time that sentinels are most apt to slumber at their post, and consequently attacks upon camps or cities, made with the intention of effecting a surprise, are usually undertaken abou this period of the morning. How well marked is the periodic tendency to sleep at this hour in all patients laboring under hectic fever produced by whatever cause. How often do we hear the poor sufferer complain of restlessly tossing about in his bed until three or four o'clock in the morning, when at last sleep, welcome although uneasy, for a few hours separates the patient from his pains. If given at an early hour in the evening, the effect of the opiate is not coincident with this periodic attempt of the constitution, and it fails in producing sleep, but if exhibited at a late hour, it begins to produce its soporific effect at the very time when nature inclines the harassed sufferer to repose, and the result of these combined influences is a deep, tranquil, and refreshing sleep. By observing this simple rule, I have often succeeded in producing sleep in cases where various narcotics had not only failed, but even added considerably to the irritation and discomfort of the patient.

In cases of sleeplessness, where you have administered an opiate with effect, be careful to follow it up for some time, and not rest satisfied with having given a momentary check to the current of morbid action. To arrest it completely, you must persevere in the same plan of treatment for a few days, until the tendency to sleep at a fixed hour becomes decidedly established. You must give an opiate the next night and the night after, and so on for five or six nights in succession, and where the watchfulness has been of an obstinate and persistent character, narcotics must be employed even for a longer period and in undiminished doses. I do not allude here to the sleeplessness which accompanies confirmed hectic and other incurable diseases; such cases require a particular mode of treatment, and generally call for all the varied resources of medicine. But in those instances of watchfulness, which are frequently observed towards the termination of acute diseases, it is always necessary to repeat the opiate for some time after you have succeeded in giving a check to this symptom. You need not be afraid of giving successive opiates lest the patient should become accustomed to them, and a bad habit be generated, for the rapid convalescence and renewed health, which are wonderfully promoted by securing a sound and refreshing sleep, will soon enable him to dispense with the use of opiates.

Another disease in which sleeplessness is a prominent symptom, is delirium tremens. We have had an example recently in our wards, and you have seen the means employed to overcome it. The patient came into hospital with symptoms of extreme nervous excitement and watchfulness, which had continued for some time, and were brought on, as is most commonly the case, by repeated fits of intoxication, succeeded by a pause of perfect sobriety—in Irishmen the result of necessity or accident. In this man you must have remarked the signal benefit which attended the use of a combination of tartar emetic and opium, and how rapidly the watchfulness disappeared. I shall not enter into the details at present, as I purpose to return to this subject on a future occasion.

There is, however, one form of nervous irritability, frequently observed in persons who are in the habit of drinking freely, but without running into excess, and presenting, as it were, a shadow of delirium tremens, on which I shall make a few remarks. This curious state of the nervous system is generally found to exist in men about the middle period of life, and who consume a larger quantity of spirituous liquors than they are able to bear. Such persons, without suffering in appearance, or losing flesh, get into a chronic state of disturbed health, manifested by nausea, and even dry retching, in the morning, loss of appetite, and impaired digestion; but, in particular, by a deranged and irritable state of the nervous system, and by watchfulness. This forms one of the most distressing symptoms, and the patient generally complains that he cannot get any sound and refreshing sleep, that he lays awake for hours together, and that when he slumbers his rest is disturbed by disagreeable dreams, or broken by slight noises. How are you to treat this affection? I can give you a valuable remedy for this deranged state of constitution—one which I have often tried, and which, from experience, I strongly recommend. It is a mixture, composed of tincture of Columbo, quassia, gentian, and bark—say an ounce of each; and to this is added a grain, or even two of morphia. A compound tincture, somewhat analagous to this, is much in use among military gentlemen and others, who have resided for a considerable time in the Indies, where from the heat of the climate, and the prevalence of intemperate habits, the stomach becomes relaxed and the nervous system irritable, so as to represent, in a minor degree, the symptoms which characterize delirium tremens. You perceive I combine several tonics to form this mixture, because they are well known to produce a more beneficial effect when combined than when administered singly ; and I add to these a narcotic, which has the property of allaying nervous excitement without deranging the intestinal canal. The dose of this mixture is a tea-spoonful three or four times a day, and the best time for taking it is about an hour before meals. It gradually removes the nausea and debility of stomach, lessens nervous irritability and watchfulness, and, with a proper and well regulated diet, and attention to the state of the bowels, I have seen it produce excellent effects. In such persons much benefit is derived from the use of the tepid shower bath.

Fever is another disease in which sleeplessness is a symptom, frequently of an unmanagable character, and pregnant with danger to the patient. You witnessed this in the case of the boy who lies in the small Fever Ward, next to the man who is at present laboring under general arthritis. This boy had fever of a mild description, and unattended with any bad symptoms. His case scarcely required any attention, and he had almost arrived at a state of convalescence without the aid of medicine, when he began to lose his rest, and absolutely became sleepless for several nights. I beg your attention to this case, for many reasons. In the first place you have seen that we tried many remedies without success, and afterwards fortunately hit on one which answered our purpose completely. Let us examine the nature of the medicines prescribed, and our reasons for giving them.

In the first place, we gave, as in the case of jaundice, an aperient, followed by a full dose of black drop. It failed in producing any sleep; we repeated it a second and a third time, but without the slightest benefit. I then remarked to the class, that as I had noticed the good effects resulting from a combination of tartar emetic and opium in the case of delirium tremens, where opium alone failed in procuring sleep, it would be proper to give this remedy a trial. I observed at the same time, that I was convinced that the preparations of antimony have a distinct narcotic effect, and that I had seen patients in fever whose watchfulness had been removed by antimony given in the form of tartar emetic or James's powder. I said it was my firm impression that tartar emetic, along with its other effects, exerts a decided narcotic influence on the system, and that it is this which makes it so valuable a remedy in treating the sleeplessness of fever and delirium tremens. Hence I have been in the habit of giving tartar emetic combined with opium in fever, and I must add, with very great success. Our predecessors were much in the habit of using antimonial mixtures in the treatment of fever, and they did this because they knew, by experience, that these remedies worked well. It is at present too much the fashion to decry their practice, and in this instance I think with very little justice.

In this boy's case, however, the combination of tartar emetic and opium did not succeed in producing sleep. Having thus failed in our first and second attempts, we had recourse to the liquor muriatus morphiæ, a preparation first brought into use by Dr. Cristison, and which in the form usually employed, is equal in strength to laudanum. It is an exceedingly valuable preparation for many reasons, and one which has the strongest claims to your notice. Being of the same strength as laudanum, it saves the trouble of learning and remembering new doses, and, in addition to this, it possesses the more important advantages of inducing sleep with more certainty, and not acting as an astringent on the bowels, or affecting the head so frequently as laudanum. You observe that I say *so frequently*; I do so because cases now and then occur in which even moderate doses of the liquor of the muriate of morphiæ produce quite as much head-cache as laudanum. I prescribed the former in doses of fifteen drops every six hours, so as to give sixty drops in the day, and continued this practice for two days, but without the slightest effect. Here you see three modes of inducing sleep completely failed. The boy remained for a day without taking any medicine, and then we made another attempt, which was more successful. We first prescribed a purgative enema, and, after this had operated, he was ordered an opiate injection, consisting of four ounces of mucilage of starch, and half a drachm of laudanum. He fell asleep shortly after using the opiate injection, and did not awake until the next morning. The following night the opiate was repeated in the same form and with equal success; convalescence went on rapidly, and the boy's health is now quite re-established.

Here, then, is a singular fact, attested by this case, that opiates in the form of injection will succeed in producing sleep, where they have completely failed when administered even in large and repeated doses by the mouth. Baron Dupuytren was the first who made this important observation, and proved that narcotics applied to the mucous surface of the rectum exercise a powerful influence on the nervous system, always equal, and very often superior, to the effect produced by taking them into the stomach. He maintains, that in delirium traumaticum and delirium tremens a certain quantity of opium, when prescribed in the form of enema, will act with more decided effect in allaying nervous excitement, than the same or even a larger quantity when taken by the mouth. I have no hesitation in giving full credit to this assertion, as the results of my experience tend strongly to confirm its truth. I have, not long since, published in the *Dublin Med. Journal*, the case of a patient in Sir P. Dun's Hospital, who was reduced to the last stage of debility and emaciation from the combined effects of mercury and syphilis. The torture which this man indured from nocturnal pains and a total deprivation of sleep, was such that he swallowed enormous doses of opium; in fact, he had, previously to his admission into Sir P. Dun's Hospital, exhausted all his means in purchasing opium. While in hospital he used to take 150 drops of black drop in the course of a day, and yet, notwithstanding these excessive doses, he could only get a few minutes of unrefreshing slumber. After some time I changed the plan of treatment, and had the black drop administered in the form of enema. It succeeded in producing a decided soporific effect, and in a short time he was able to enjoy a sufficient quantity of repose, from taking only one-tenth of the quantity used by the mouth. I have also, in the same paper, adverted to the case of a medical gentleman who labored under an affection of his joints, which was accompanied by spasms of the limbs, and most excruciating pains. His agony was so intense that he used to swallow grain after grain of opium, until he had taken to the amount of thirty or forty grains, with the view of procuring some alleviation of his sufferings. He was prevailed on to give up altogether the use of opium by the mouth, and employ it in the form of enema, which he did with the most striking advantage, the quantity which succeeded in giving relief in this way being scarcely the twentieth part of what he ordinarily used.

It is unnecessary for me to enter here into any discussion with respect to the nature and treatment of delirium traumaticum, and the sleeplessness which always accompanies it, as you will find this subject very ably treated in M. Dupuytren's works, and in a very instructive and elegant lecture delivered by Mr. Crampton (the Surgeon General) in this hospital, and published in the last volume of the *London Medical and Surgical Journal.* There is, however, one kind of sleeplessness arising from irritation of the skin produced by blisters, which frequently assumes a very serious character, and on which it may be necessary to offer a few observations, as the subject has not been noticed sufficiently by practical writers. Trifling as the irritation resulting from a blister may seem, yet, under certain circumstances, it is a symptom of highly dangerous aspect, and becomes a source of just alarm. I have witnessed the loss of some lives from this cause, and many patients have, to my knowledge, been rescued from impending danger, by an early and proper share of attention being directed to its phenomena and treatment.

The bad effects on the nervous system occasionally produced by the application of blisters, are somewhat analogous to those which result from wounds and other external injuries, and to be accounted for on the same principle. Wounds and injuries sometimes make an impression on the nervous system, by no means proportioned to the importance of the injured organ to life, or to the extent of the mischief. An injury produced by a body which strikes the sentient extremities of the nerves with great force, will sometimes produce very remarkable effects on the system. Thus a musket ball striking a limb may, without wounding any great artery or nerve, or destroying any part of importance to life, produce a train of nervous symptoms of an extraordinary character. The person, without feeling much pain, and scarcely knowing that he has been wounded, without being terrified, or having his imagination excited by any apprehended dangers, turns pale, gets a tendency to faint, and sometimes actually dies from the impression made on the nervous system. In the same way an external injury re-acting on the nerves may bring on high mental excitement, delirium, and a total privation of sleep, as we exemplified in delirium traumaticum. I mention this with the view of establishing the proposition that impressions made on the sentient extremities of the nerves are sometimes reflected on the nervous centres, producing the most alarming effects. In this way we can understand how the irritation of blisters may produce sleeplessness, mental aberration, and a train of symptons analogous to those which characterize delirium traumaticum.

The delirium and sleeplessness arising from the irritation of blisters is by no means an uncommon disease. I have seen many examples of it in private practice, and I am anxious that you should be acquainted with its nature and treatment. It is generally met with in the case of children, in whom the cutaneous surface is extremely tender and irritable. I could relate several instances in which I have been called on to visit children laboring under fever, where symptoms of high nervous excitement were present, and where I found the little patients delirious, screaming, and perfectly sleepless from this cause. I have found this alarming affection generally occurring at an advanced stage of fever, and exhibiting a train of symptoms which closely resemble hydrocephalus. I have observed that after the application of a blister to relieve some suspected cerebral, or abdominal, or thoracic affection, jacitation, restlessness, constant application of the hand to the head, and delirium have appeared, and that these symptoms had been mistaken for incipient cerebritis or hydrocephalus, and treated with leeches and purgatives. When the blister had been applied to the nape of the neck, the soreness and irritation of the skin on that part *cause the child to roll its head from side to side on the pillow, with that peculiar motion and scream supposed to prove to a demonstration the existence of hydrocephalus.* I have learned also, that the above measures, so far from giving relief, have only tended to produce an exacerbation of the disease, and that the medical attendant has given up the case in despair. Now, gentlemen, if called to such a case what should be your practice? In four cases of this kind I gave my opinion frankly to the medical attendant, and told him he was pursuing a wrong course, that the disease was analagous to delirium traumaticum, and not to be treated by leeches or purgatives, and least of all by blisters. I observed to him that these symptoms had made their appearance shortly after the child had been blistered for suspected disease of the belly, or head, or chest; and that it was useless to attempt to remove the disease by leeches, or purgatives, or blisters. The remedy I always proposed was opium, and it was acknowledged in four or five cases, that this remedy had succeeded, not merely in relieving the existing symptoms, but in saving the patient's life. In such cases, particularly in young children, the opium must be given in small but frequently repeated doses, so as to ensure its energetic, but safe action, and the greatest care must be taken to soothe the irritated portion of the skin, by ointments, poultices, &c., *while unwearied diligence must be bestowed upon the task of preventing the child from scratching the blistered surface.* To effect this the child's hands must be muffled in appropriate gloves, and must be secured in the sleeves of a shirt made for the purpose.

I beg your attention still further to this subject of sleeplessness and delirium. I wish to mention the case of a gentleman who was a pupil of mine. This gentleman studied hard, attended lectures regularly, and was constantly in the dissecting room. While thus occupied, he happened to wound one of his toes in paring a corn, and afterwards wore a tight shoe on the injured foot. A small imperfect abscess formed in the situation of the corn, which was opened by one of his fellow students; the incision gave very great pain, and was not followed by any discharge of matter. Next day he was feverish, and the lymphatics of the injured limb became extensively engaged, the inflammation ascending towards the glands of the groin, and having a tendency to form a chain of insulated patches in different parts of the leg and thigh along the course of the lymphatics. This you will generally find to be the case in inflammatory affections of the lymphatics, the inflammation is seldom continuous, but in the majority of cases, is developed at certain insulated points, where small diffuse suppurations form very rapidly. After a few days, this young gentleman's fever increased to an alarming height; he became completely sleepless, and had incessant delirium. He was purged briskly, leeched extensively and repeatedly, his head shaved, and cold applications so constantly applied, that he appeared half drowned and collapsed. Notwithstanding this very active treatment, not the slightest relief was obtained; neither were the symptoms mitigated by incisions made in the inflamed patches for the purpose of evacuating matter; the sleeplessness continued, and the delirium was as wild as ever. I saw him on the seventh or eighth day, when all antiphlogistic measures had failed, and his friends were quite in despair. On being asked my opinion, I stated that I looked upon the case as one of delirium, not proceeding from any determination to the head or inflammation of the brain, but depending on a cause analagous to those which produce delirium traumaticum, and that instead of antiphlogistics I would recommend a large dose of opium and some porter to be immediately given. Mr. Cusack, who visited the patient after me, concurred in this view, and a full opiate was administered in repeated doses. It succeeded in producing sleep and tranquilizing the nervous excitement. I may here observe that a few days afterwards this gentleman had a return of the symptoms of cerebral disturbance with sleeplessness, in consequence of omitting his opiate, and that the opiate and porter were again administered, and again succeeded in removing the delirium and watchfulness. By perseverance in the use of the same means, the disease was completely removed, and convalescence established.

The last kind of sleeplessness to which I shall direct your attention, is that which is frequently met with in persons of a nervous and irritable disposition, in hypochondriacs, and hysterical females. You find such persons, although of active habits, and with tolerable appetites, complaining of a total privation of their natural rest, and it is astonishing to think how long they may continue subject to this harassing watchfulness. I have frequently observed this affection among females of nervous habit, who possessed strong feelings of attachment to the interest and welfare of their families, and who were remarkable for an exemplary and over anxious discharge of their domestic duties. It is also very often met with in the upper classes of life, where the susceptibility to nervous excitement is morbidly increased by fashionable habits. I shall not enter into the various moral causes which tend to produce this state of the nervous system, and will content myself for the present with giving you some hints for the treatment of this obscure affection. As yet I have not any distinct and accurate notions of the disease, and can only guess at the treatment, but this much I may state, that such cases are not to be cured by the means which I have already detailed. If they are to be cured by any means, I think it is by antispasmodics, and remedies which have a gently stimulant, and, if I may so express myself, alterative effect on the nervous system. I have cured two cases of this kind by musk and assafœtida, where every other remedy had failed. To one of these I was called by my friend, Dr. Neason Adams; the patient was a lady of delicate constitution and hysterical habit; she was emaciated, and suffered from a total loss of rest, but had no other disease. All kinds of narcotics had been tried unsuccessfully, and opium in all its forms had failed in procuring sleep. I advised the use of musk in doses of a grain every second hour, and this means proved eminently successful. In another case I succeeded by administering the same remedy in combination with assafœtida. I have also remarked that assafœtida alone, given in doses of two or three grains three times a day, has very considerable effect in calming nervous irritation of this description, and restoring the patient to the enjoyment of more prolonged and refreshing sleep. In all such cases the physician must be most careful to have the appearance of not thinking the loss of sleep as a matter of much consequence, and the family of the patient must be directed to speak as little about the matter in his presence as possible,—nay, so powerful is the operation of moral impressions, that in one case which I attended along with Mr. Halahan, I succeeded in procuring sleep by ordering a musk pill to be given every second hour, night and day, and by desiring the patient to be awakened, should she be asleep, at the time the pill was to be taken. I laid great stress on the importance of so proceeding, and thereby produced so strong an effect on the patient's mind, and inspired so great a confidence in the efficacy of the medicine, that she went to bed, not so much afraid of lying awake as afraid of being asleep at the hours when she ought to take a pill. The idea which had hitherto fixedly occupied her mind was displaced by a new impression, and relief was obtained the very first night.

To conclude, gentlemen, I may observe that sleeplessness in a chronic form is often produced by dyspepsia, and can only be relieved by the means suited to indigestion. Here it is that small doses of blue pill and tonic purgatives are of infinite service, combined with change of air, of scene, and an appropriate diet. In many females, sleeplessness is combined with menstrual irregularity, and can only be cured by means calculated to invigorate the health and restore the catamenial discharge to its natural periods and quantity, for the nervous system suffers equally whether they be suppressed or overabundant. It is singular how long sleeplessness often continues in chlorosis without inducing those serious consequences that are produced by this symptom in other morbid states of the system. In such cases much is sometimes accomplished by means of the common preparations of morphia, or by the use of Hoffman's (liquor æthereus oleosus,) camphor, and other medicines that act upon the nervous system. It must be confessed, however, that these and every other expedient to obtain sleep often fail in chlorotic and hysterical females, in whom relief is only obtained by a gradual improvement of the general health and menstrual function.—*London Med. and Surg. Jour.*, 21*st March*, 1835.—*Ibid.*

